# Molecularly Woven Cationic Covalent Organic Frameworks for Highly Selective Electrocatalytic Conversion of CO_2_ to CO

**DOI:** 10.1002/advs.202408152

**Published:** 2024-09-10

**Authors:** Fentahun Wondu Dagnaw, Karim Harrath, Tao Zheng, Xu‐Dong Wu, Yu‐Ze Liu, Rui‐Qi Li, Luo‐Han Xie, Zhen Li, Xuezhong He, Qing‐Xiao Tong, Jing‐Xin Jian

**Affiliations:** ^1^ Department of Chemistry Key Laboratory for Preparation and Application of Ordered Structural Materials of Guangdong Province and Guangdong Provincial Key Laboratory of Marine Disaster Prediction and Prevention Shantou University Guangdong 515063 P. R. China; ^2^ Department of Chemistry Southern University of Science and Technology Shenzhen 518055 P. R. China; ^3^ Fundamental Science Center of Rare Earths Ganjian Innovation Academy of Sciences Ganzhou 431000 P. R. China; ^4^ Department of Chemical Engineering Guangdong Technion – Israel Institute of Technology Shantou 515063 P. R. China

**Keywords:** carbon capture, CO_2_ reduction, covalent organic frameworks, electrocatalytic, palladium nanoparticles

## Abstract

Coupling carbon capture with electrocatalytic carbon dioxide reduction (CO_2_R) to yield high‐value chemicals presents an appealing avenue for combating climate change, yet achieving highly selective electrocatalysts remains a significant challenge. Herein, two molecularly woven covalent organic frameworks (COFs) are designed, namely CuCOF and CuCOF^+^, with copper(I)‐bisphenanthroline complexes as building blocks. The metal–organic helical structure unit made the CuCOF and CuCOF^+^ present woven patterns, and their ordered pore structures and cationic properties enhanced their CO_2_ adsorption and good conductivity, which is confirmed by gas adsorption and electrochemical analysis. In the electrocatalytic CO_2_R measurements, CuCOF^+^ decorated with extra ethyl groups exhibit a main CO product with selectivity of 57.81%, outperforming the CuCOF with 42.92% CO at the same applied potential of 0.8 V_RHE_. After loading Pd nanoparticles, CuCOF‐Pd and CuCOF^+^‐Pd performed increased CO selectivity up to 84.97% and 95.45%, respectively. Combining the DFT theoretical calculations and experimental measurements, it is assumed that the molecularly woven cationic COF provides a catalytic microenvironment for CO_2_R and ensures efficient charge transfer from the electrode to the catalytic center, thereby achieving high electrocatalytic activity and selectivity. The present work significantly advances the practice of cationic COFs in real‐time CO_2_ capture and highly selective conversion to value‐added chemicals.

## Graphical abstract

A molecularly woven cationic COF provides a catalytic microenvironment for CO_2_R and ensures efficient charge transfer from the electrode to the catalytic center, thereby achieving high electrocatalytic activity and high CO production selectivity of 94.45%.

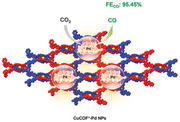

## 1 Introduction

The increasing emission of CO_2_ into the atmosphere due to the massive consumption of fossil fuels poses an alarming threat to the world.^[^ ^1^ ^]^ Therefore, developing efficient and highly selective carbon capture, storage, and conversion technologies is becoming an urgent issue.^[^ ^2^ ^]^ The ideal blueprint of CO_2_ conversion is inspired by natural photosynthesis, which perfectly utilizes solar energy to convert CO_2_ and water into glucose.^[^ ^3^ ^]^ Currently, a variety of systems have been developed, such as electrochemical,^[^ ^4^ ^]^ photochemical,^[^ ^5^ ^]^ photovoltaic electrochemical,^[^ ^6^ ^]^ and photoelectrochemical systems,^[^ ^7^ ^]^ to capture and convert CO_2_ into valuable chemical products. To accomplish an efficient and selective CO_2_ conversion, the development of newly functionalized materials with improving the CO_2_ adsorption, activation, and catalytic conversion capabilities is regarded as the key part of CO_2_ conversion.

Covalent organic frameworks (COFs), a class of crystalline porous materials, whose overlaying stacking and long‐range order show outstanding characteristics in optical and electrical aspects,^[^ ^8^ ^]^ can provide a favorable microenvironment for CO_2_ capture and reduction.^[^ ^9^ ^]^ By adjusting the cavity size and introducing specific functional groups of COFs, their selective capture and separation of CO_2_ could be regulated.^[^ ^10^ ^]^ Currently, artificial photocatalytic systems are constructed by COFs to simulate natural photosynthesis for the CO_2_ conversion and utilization of solar energy.^[^ ^2^, ^11^ ^]^ However, due to the limited charge transport properties of the organic structure, the traditional COF material has poor conductivity and few active sites, which limits their application in electrocatalytic systems. Therefore, the introduction of transition metals coordination and cationic functional group (cationic COFs) is an effective strategy to enhance its conductivity and CO_2_ adsorption capacity toward CO_2_ conversion.^[^ ^12^ ^]^

Recently, O. M. Yaghi's group just reported a kind of 2D molecular weaving COFs using aldehyde‐functionalized copper(I) bisphenanthroline complex as cross‐linking points, but its further catalytic reduction properties for CO_2_ capture and conversion had not been reported yet. Copper complexes were widely used as CO_2_R catalysts to achieve hydrocarbons,^[^ ^13^ ^]^ but drawbacks by their poor selectivity, low durability, and high overpotentials. Meanwhile, palladium (Pd)‐based nanomaterials have attracted considerable attention in CO_2_RR, for which enables high selectivity to produce CO, or formic acid (HCOOH) at different voltages.^[^ ^14^ ^]^ Unfortunately, the strong surface affinity of Pd for CO often leads to the deactivation of CO_2_ to CO conversion, and the aggregation and size growth of Pd nanoparticles (NPs) reduce their catalytic activity sites.^[^ ^15^ ^]^ Consequently, great challenges still remain in preparing uniformly dispersed Pd NPs and regulating their catalytic microenvironments. Herein, cationic COFs could serve as a protective layer of Pd NPs to make them uniformly dispersed, providing a microenvironment to facilitate CO_2_ adsorption, activation, and catalytic processes, thereby achieving efficient and highly selective CO_2_ reduction.

**Scheme 1:**
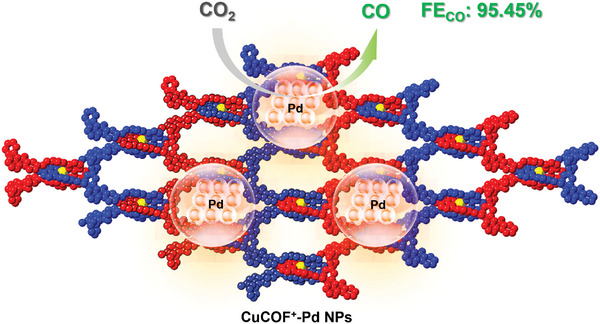
Molecularly woven COFs with Pd NPs for highly selective CO_2_‐to‐CO conversion.

In this work, two novel COFs with copper‐coordinated structures, namely CuCOF and cationic CuCOF^+^, are prepared as CO_2_RR catalysts (**Scheme** **1**). The metal–organic helical structure unit makes the CuCOF and CuCOF^+^ present woven patterns, and their ordered pore structures and ionic properties enhance their CO_2_ adsorption and good conductivity, which is confirmed by gas adsorption and electrochemical analysis. In the electrochemical CO_2_RR measurements, highly selective CO_2_‐to‐CO conversion is achieved with Faraday efficiency over 90%. DFT theoretical calculations and experimental measurements have verified the positive charge properties and the influence of Pd NPs on the CO_2_RR mechanism.

## 2 Results and Discussion

### 2.1 Synthesis and Characterization of CuCOFs

**Figure 1:**
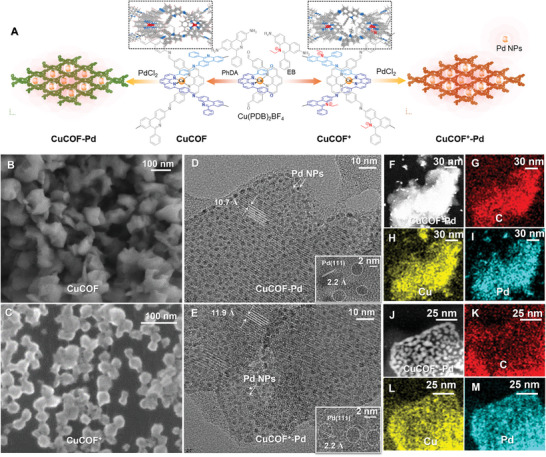
A) Synthesis routes of CuCOF, CuCOF^+^, CuCOF‐Pd and CuCOF^+^‐Pd catalysts. SEM images of CuCOF (B) and CuCOF^+^ (C); HRTEM images of CuCOF‐Pd (D) and CuCOF^+^‐Pd (E), inset figure shows the lattice of COFs and Pd NPs; HAADF‐EDS images of CuCOF (F–I) and CuCOF^+^ (J–M).

Two new molecularly woven COF materials, namely CuCOF and CuCOF^+^, are synthesized by the acid‐catalyzed Schiff‐base condensation reaction between Cu(PDB)_2_ and 6‐phenylphenanthridine‐3,8‐diamine (PhDA) and 3,8‐diamino‐5‐ethyl‐6‐phenylphenanthridin‐5‐ium bromide (EB), respectively (**Figure** **1**A). The detailed synthesis routes and conditions for CuCOFs are given in Supporting Information (Schemes S1–S3, Figures S1 and S2, Supporting Information). According to the reported works on 2D woven COF,^[^ ^16^ ^]^ the prepared CuCOF and CuCOF^+^ are characterized by Fourier transform infrared spectroscopies (FTIR), powder X‐ray diffraction (PXRD), and thermogravimetric analysis (TGA). As depicted in Figure S3 (Supporting Information), precursors of PhDA and EB display their N─H stretching vibration (ν_N‐H_) peaks at the region of 3300–3400 cm^−1^, and aromatic C─H stretching vibration (ν_Csp2‐H_) peaks at the region of 3000–3100 cm^−1^, and the typical stretching vibration of ethyl group (‐CH_2_CH_3_) at 2856 and 2921 cm^−1^. Meanwhile, the stretching vibration of C═O in the precursor of [Cu(PDB)_2_]BF_4_ presents at 1700 cm^−1^, which disappeared in the prepared CuCOF and CuCOF^+^, implying that the aldehyde group on the Cu‐complex are fully converted into a C═N structure (1650–1620 cm^−1^). PXRD is performed to investigate the crystallinity of CuCOF and CuCOF^+^. In the PXRD pattern, CuCOF displays an extremely strong edge diffraction peak at 3.65° and weak diffraction peaks at 6.60° and 8.30°, while CuCOF^+^ exhibits peaks at 3.63°, 6.72°, and 8.20° (Figure S4, Supporting Information). PXRD pattern confirm the present of diffraction peaks of Pd(020), Pd(111), PdO(100) and PdO(101) in both CuCOF‐Pd and CuCOF^+^‐Pd. A structural model of CuCOF is established in the *P222* orthogonal space group (Figure S5, Supporting Information),^[^ ^16b^ ^]^ and the simulated PXRD was aligned with the experimental pattern. According to the refined model, CuCOF^+^ crystallizes in a chain‐link fence pattern of covalently linked 1D helical threads to construct a 2D pore system with channels.

The Brunauer–Emmett–Teller (BET) surface areas and pore width distributions of the CuCOF and CuCOF^+^ are determined by N_2_ adsorption‐desorption measurements at 77 K (Figure S6A, Supporting Information). Accordingly, CuCOF presents a larger BET surface area of 38.6079 ± 1.0553 m^2^ g^−1^ than that of CuCOF^+^ (27.6426 ± 0.8744 m^2^ g^−1^), implying the decorated ethyl groups in CuCOF^+^ occupy its pore space (Table S1, Supporting Information). Moreover, the Barrett–Joyner–Halenda (BJH) desorption pore size distributions of CuCOF and CuCOF^+^ shows mesopores structure with diameters of ≈3.12 and 2.80 nm, respectively, which is consistent with the pore sizes of 3.03 and 2.80 nm in the theoretical calculation models (Figure S6B,C, Supporting Information). Interestingly, the BET surface area of CuCOF‐Pd and CuCOF^+^‐Pd decreased sharply after loading Pd NPs, which are only 7.0386 ± 0.2025 and 4.0780 ± 0.0064 m^2^ g^−1^ (Table S1, Supporting Information), respectively, and the mesopores structures are also significantly reduced (Figure S6A, Supporting Information). These results indicate that Pd nanoparticles exist in the pores of COFs, and their size should be smaller than the pore size.

Moreover, the CO_2_ adsorption capacities of CuCOF and CuCOF^+^ are identified by BET CO_2_ adsorption‐desorption measurements at 273 and 298 K, respectively (Figure S7, Supporting Information). Hence, the CO_2_ adsorption capacities of CuCOF and CuCOF^+^ are found to be 11.23 versus 6.97 cm^3^ g^−1^ at 273 K, and 6.48 versus 4.27 cm^3^ g^−1^ at 298 K, respectively. The higher CO_2_ adsorption capacity of CuCOF over CuCOF^+^ is related to its larger surface area and the stronger basicity of heteroatom N, which can contribute to the chemical adsorption of CO_2_. The thermal stability is studied by thermogravimetric analysis (TGA) measured under an N_2_ atmosphere, and the onset of the thermal decomposition of CuCOF is found to be at 462 °C (Figure S8, Supporting Information), aligning with previously reported waving COFs.^[^ ^16^, ^17^ ^]^ In contrast, CuCOF^+^ displays weight loss at 214 and 490 °C, the initial weight loss process might be attributed to the removal of ethyl bromide.

Afterward, Pd NPs are deposited on the COFs using PdCl_2_ as the precursor. The surface morphology of CuCOFs and COFs‐Pd are characterized by scanning electron microscopy (SEM) and transmission electron microscopy (TEM). The CuCOF^+^ presents a size distribution in the range of 20–30 nm, which is relatively smaller than the CuCOF of 50–100 nm (Figure 1B,C). High‐resolution TEM is conducted to explore the additional insights into the crystal structure of COFs and Pd NPs, which have clear lattice spacing of 10.7, 11.9, and 2.2 Å, respectively (Figure 1D,E). These fringes correspond to the (001) lattice plane of COFs,^[^ ^16b^ ^]^ and the (111) lattice plane of Pd NPs.^[^ ^14^ ^]^ The dense charge distribution of CuCOF^+^ leads to a loose woven structure, which is consistent with the large lattice space observed by HRTEM. Interestingly, Pd NPs on CuCOF and CuCOF^+^ are uniformly dispersed along the lattice fringes of COF, with similar sizes of 2.0–2.5 nm (Figure 1D‐E). Combined with the analysis result of BJH desorption pore size distributions, it is proved that Pd NPs are evenly distributed in the pore size of COFs. The isolated Pd NPs provide sufficient surface area and binding sites to ensure high CO_2_R activity. High‐angle annular dark‐field energy dispersive spectroscopy (HAADF‐EDS) is carried out to identify the distribution mappings of C, Cu, and Pd elements, confirming the successful formation of CuCOF‐Pd (Figure 1F–I) and CuCOF^+^‐Pd (Figure 1J–M). Besides, contact angle measurements are performed to distinguish the wettability of CuCOF and CuCOF^+^ with and without loading Pd NPs. As shown in Figure S9 (Supporting Information), CuCOF^+^ reveals a smaller contact angle of 70.98° than that of CuCOF (84.69°), indicating that the modification of cationic functional groups could improve the hydrophilicity of CuCOF. Furthermore, the decoration of Pd NPs onto CuCOF and CuCOF^+^ could further enhance their hydrophilicity with significantly reduced contact angles of 65.99° and 62.41°, respectively. A hydrophilic material could promote the free passage of CO_2_ substrate and protons on the electrode–electrolyte interface and achieve efficient CO_2_R performance.

### 2.2 Electrocatalytic CO_2_R Performance

**Figure 2:**
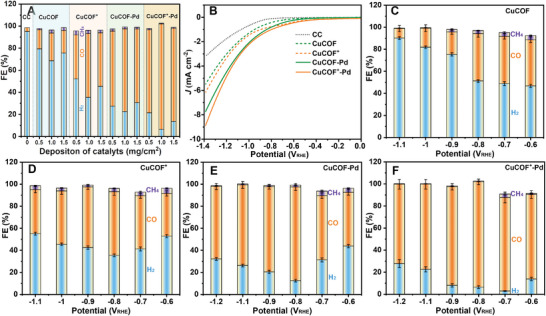
A) Faradaic efficiency of gas products using CC electrode and with different deposition amounts of CuCOF, CuCOF^+^, CuCOF‐Pd and CuCOF^+^‐Pd catalysts. LSV of CuCOF and CuCOF+ with and without Pd NPs (B). Faradaic efficiency of CuCOF (C), CuCOF^+^ (D), CuCOF‐Pd (E) and CuCOF^+^‐Pd (F) at different applied potentials ranging from −0.6 to −1.2 V_RHE_.

To evaluate the electrocatalytic CO_2_R performance in a customized H‐cell, CuCOF and CuCOF^+^, CuCOF‐Pd, and CuCOF^+^‐Pd electrocatalysts are deposited on the pretreated carbon cloth (CC) electrode. A custom‐made H‐cell consisting of a proton exchange membrane (Nafion‐117) to separate the anode (Pt electrode) and cathode (working electrode), is filled with 60 mL of 0.1 m potassium carbonate (KHCO_3_) aqueous solution as the electrolyte. The half‐cell with the working electrode is continuously bubbling with high‐purity CO_2_ at a flow rate of 30 sccm, and is connected to the online gas chromatography (GC‐7920) equipped with a flame ionization detector (FID) and thermal conductivity detector (TCD). Three gaseous H_2_, CO, and CH_4_ products are detected, and the total Faraday Efficiency (FE) is close to 100% attachment. No obvious liquid product is detected in the proton nuclear magnetic resonance (^1^H‐NMR) spectroscopy of the electrolyte even after long‐time electrolysis. Without loading of the COFs catalysts, the bare CC electrode at −0.8 V_RHE_ exhibits a low current density of ≈−0.1 mA cm^−2^ and a major product of H_2_ with FE(H_2_) over 95.32% (**Figure** **2**A). After loading 0.5, 1.0, and 1.5 mg cm^−2^ of the COFs catalysts, their current densities are significantly increased with releasing of CO_2_R product, mainly CO. At the optimal catalyst loading of 1.0 mg cm^−2^, the selectivity of CO production gradually increased from 3.50% without a catalyst to 42.92, 57.81, 84.97, 95.45% for CuCOF, CuCOF^+^, CuCOF‐Pd, and CuCOF^+^‐Pd catalysts, respectively. Among them, the ethyl‐modified CuCOF^+^ and CuCOF^+^‐Pd have relatively higher CO selectivity than their counterparts of CuCOF and CuCOF‐Pd, and the optimal CuCOF^+^‐Pd is 27.3 times of the catalyst‐free CC electrode. To the best of our understanding, we speculate that the positive charge modification of COF materials is beneficial to overcome the side reaction of HER, which could inhibit the transport of protons to the electrode surface via electrostatic repulsion (Table S2, Supporting Information).^[^ ^18^ ^]^

The electrocatalytic CO_2_R current increases with the introduction of COF catalysts in the region −0.4 to −1.4 V_RHE_ (Figure 2B). In Tafel plots (Figure S10, Supporting Information), the onset potential (E_on_) to achieve a current density of −0.1 mA cm^−2^, is gradually decreased from −0.79 V_RHE_ for the CC electrode to −0.09 V_RHE_ for the CuCOF^+^‐Pd electrode. According to the Faraday efficiency distribution of the gas products at different voltages, all COF‐loaded electrodes have a maximum CO selectivity at −0.8 V_RHE_, and uninhibited H_2_ products at lower or higher voltages (Figure 2C–F). In the Tafel plots, these electrodes present different slopes at potentials −0.5−0.8 V_RHE_ and above −1.0 V_RHE_, indicating that the CO_2_R has multiple kinetic processes on the electrode surface. More interestingly, the Tafel slopes in the region below −0.8 V_RHE_ gradually increase from 236 mV dec^−1^ of bare CC electrode to 376, 370, 468, and 515 mV dec^−1^ with the loading of CuCOF, CuCOF^+^, CuCOF‐Pd, and CuCOF^+^‐Pd catalysts, and decrease from 648 mV dec^−1^ to 625, 601, 587, and 583 mV dec^−1^ in the region above 1.0 V_RHE_ (Figure S10, Supporting Information). Since the CO_2_‐to‐CO conversion requires the consumption of protons while avoiding the reduction of protons to produce H_2_, it is necessary to construct a high‐selectively catalytic microenvironment to balance CO_2_R and HER. The cationic COF materials based on the copper complex inhibit the proton transport to the electrode surface at low potentials and increase their Tafel slopes. As the potential rises to ≈−0.8 V_RHE_, the CO_2_R and proton transport achieve the best balance to obtain the highest CO selectivity. When the voltage progress increases to over 1.0 V_RHE_, the contribution of proton reduction increases with producing H_2_, which is consistent with the Faraday efficiency results.

### 2.3 Stability of CuCOFs‐Pd for CO_2_R

**Figure 3:**
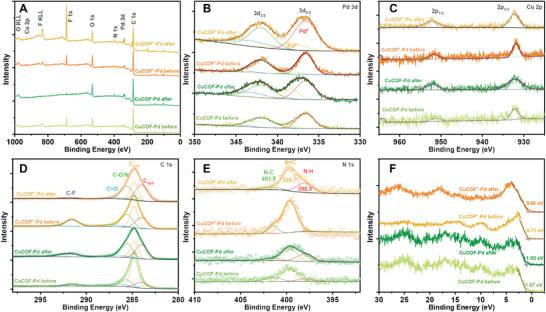
A) Survey XPS of CuCOF‐Pd and CuCOF^+^‐Pd before and after CO_2_R. High‐resolution XPS of Pd 3d (B), Cu 2p (C), C 1s (D), N 1s (E). F) Difference of valence band and Femi energy (*E*_VB_−*E*_F_).

In continuous electrocatalytic CO_2_R, CuCOF‐Pd, and CuCOF^+^‐Pd maintain 80–85% and 88–95% of CO product selectivity at −0.8 V_RHE_ within 210 min, respectively (Figure S11, Supporting Information). To investigate the elemental compositions and oxidation states of CuCOF‐Pd and CuCOF^+^‐Pd, X‐ray photoelectron spectroscopy (XPS) analysis was performed before and after CO_2_R measurements (**Figure** **3**). In survey XPS, CuCOFs‐Pd exhibited similar elemental peaks of C_1s_, N_1s_, Pd_3d_, and Cu_2p_ before and after CO_2_R (Figure 3A), implying their high stability during CO_2_R. As shown in Figure 3B, the high‐resolution Pd 3d spectra display two spin‐orbit doublets, which are assigned to Pd^0^ and Pd^2+^ with characteristics of Pd 3d_5/2_ (342.1 and 343.6 eV), Pd 3d_3/2_ (336.7 and 338.3 eV). After electrocatalytic CO_2_R, the peaks of Pd 3d_5/2_ in CuCOF‐Pd and CuCOF^+^‐Pd have slightly shifted to high binding energies, indicating that the Pd provides a catalytic site for CO_2_R and coordinates with the intermediates. High‐resolution XPS Cu 2p spectra confirm the presence of Cu^+^ with typical peaks of Cu_2p3/2_ and Cu_2p1/2_ at 932.2 and 951.1 eV, respectively (Figure 3B). After CO_2_R, the binding energy of Cu 2p peaks are anchored at the same positions, confirming their high stability Cu‐based COFs (Figure 3C). High‐resolution XPS C1s displays the fitted peaks at binding energies of 283.0, 284.8, 285.6, and 291.6 eV, corresponding to the C_sp2_, C_sp3_(C─C), C─O/C─N, and C─F groups, respectively (Figure 3D). After the CO_2_R measurement, the area ratios of C_sp2_ and C─O/N fitted peaks increased, which was speculated to be related to the adsorbed carbonate. Moreover, high‐resolution N1s XPS spectra showed fitted peaks at 397.9, 399.7, and 401.6 eV, assigning to N─H, N═C, and N─C groups, respectively (Figure 3E). The enhanced N─H signal is attributed to the partial hydrolysis of the Schiff base C═N group during the CO_2_R, which was confirmed by FTIR spectra of CuCOF^+^ after long‐time stability measurements (Figure S12, Supporting Information). Additionally, the valence band (*E*_VB_) edge of the CuCOF‐Pd and CuCOF^+^‐Pd is obtained at 1.07 and 0.71 eV, respectively, when its Fermi energy (*E*_F_) is set at 0 eV (Figure 3F). Combined with their optical bandgaps obtained from the Tauc plots and UV–vis absorption spectra (Figure S13, Supporting Information), the conduction band (*E*_CB_) position of CuCOF‐Pd and CuCOF^+^‐Pd was located at −0.38 and −0.85 eV, respectively, which meets the required potential for CO_2_ reduction, and CuCOF^+^‐Pd surpass the reduction ability of the former.

### 2.4 Mechanism Insights

To further explain the effect of cationic groups on their catalytic activity, we performed the surface charge analysis of CuCOFs. Zeta potential measurements confirm that CuCOF and CuCOF^+^ has positive charges of +39.2 and +55.0 mV, respectively, in an aqueous solution (Figure S14, Supporting Information). After loading the Pd catalyst, the material was still positively charged, but the charge density decreased maybe due to the electron delocalization between CuCOFs and Pd NPs. Kelvin probe force microscopy (KPFM) analysis shows that the surface potentials of Pd NPs decorated CuCOFs increased from 13.0 ± 0.6 and 17.0 ± 0.6 mV to 46.0 ± 2.0 and 42.5 ± 0.5 mV, respectively (Figure S15, Supporting Information). The enhanced surface potential indicates that the loading of Pd NPs further bends the energy level structure of CuCOF, and its built‐in electric field facilitates charge separation and transport, thereby achieving more efficient catalytic CO_2_R performance. Electrochemical impedance spectroscopy (EIS) of CuCOFs‐Pd has demonstrated a smaller arc radius than the bare CuCOFs, corresponding to its improved charge transfer and catalytic abilities (Figure S16, Supporting Information).

**Figure 4:**
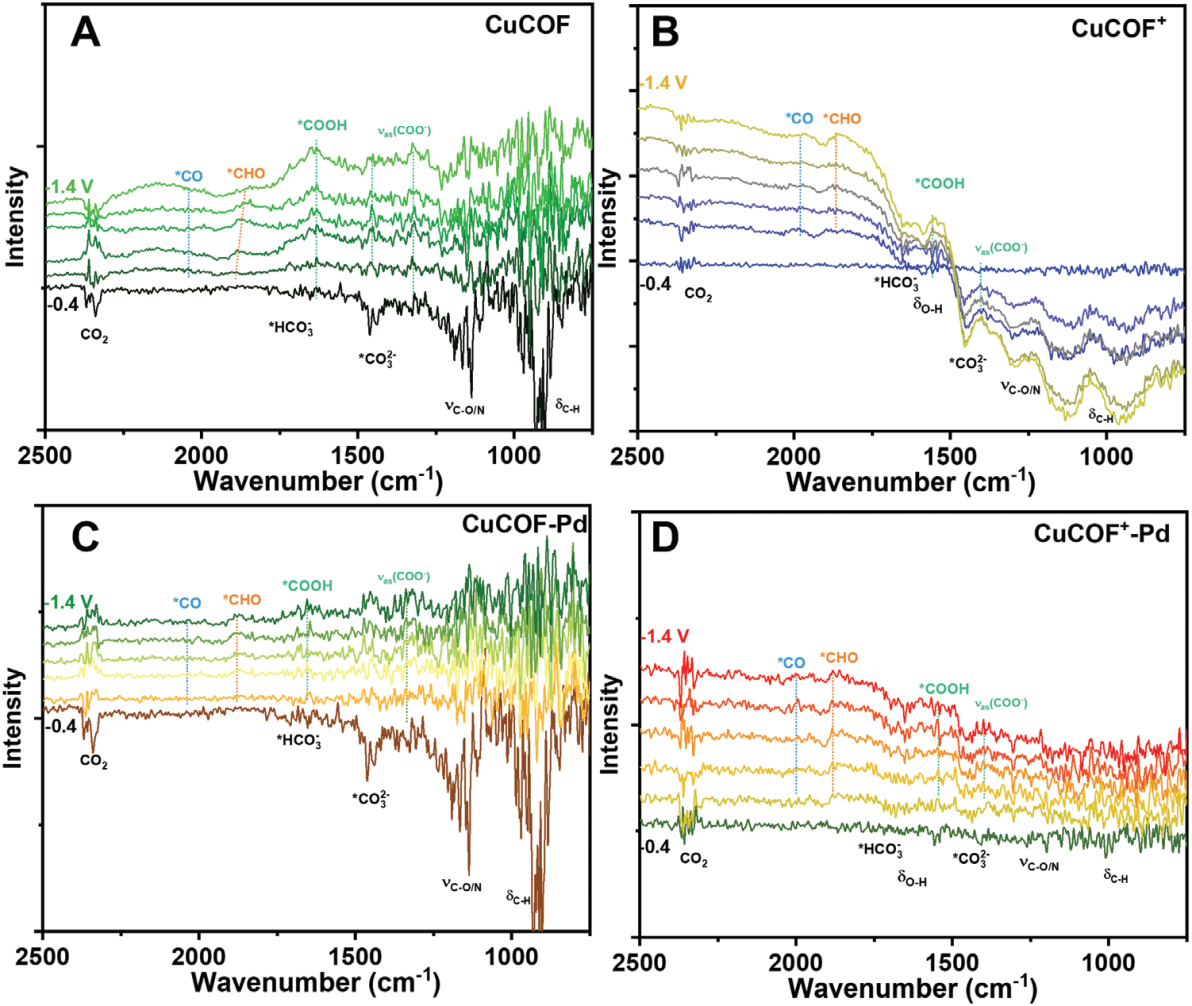
In situ ATR‐SEIRAS of CuCOF (A), CuCOF^+^(B), CuCOF‐Pd (C) CuCOF^+^‐Pd (D) at applied potentials from −0.4 to −1.4 V_RHE_.

To gain mechanism insights into catalytic CO_2_R by CuCOFs with and without Pd NPs, in situ attenuated total reflection‐surface‐enhanced infrared absorption spectroscopie (ATR‐SEIRAS) were performed to verify the reaction intermediates. As shown in **Figure** **4**A, the absorption bands located at ≈2045, ≈1880, ≈1634 cm^−1^ are assigned to the *CO, *CHO and *COOH intermediates, respectively. The bleaching signals are attributed to the characteristic signals of functional groups such as CO_2_, CO_3_^2−^, C─N/O, and C─H, which are labeled inside of figures. With the increase of applied voltage, the signals of CO_2_R intermediates increase, and the characteristic signals of CO_2_ substrates decrease, indicating that the electrode is undergoing an electrocatalytic CO_2_R process. In contrast, CuCOF^+^ has more significant bleaching signals than CuCOF, which may be attributed to the instability of the modified ethyl structure. After the loading of Pd NPs, the wavenumber of the characteristic signals of the CO_2_R intermediates shifted slightly and weakened significantly. This result indicates that Pd NPs could provide new CO_2_R reaction sites and achieve a faster catalytic cycle, resulting in a decrease in the concentration of catalytic intermediates.

**Figure 5:**
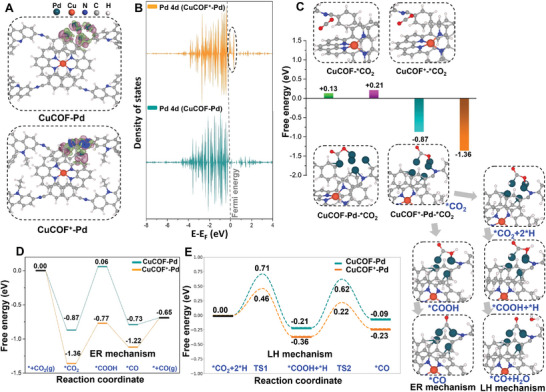
DFT calculated charge density (A) and PDOS of Pd 4d orbitals of CuCOF‐Pd and CuCOF^+^‐Pd (B). (C) CO_2_ adsorption energies and models of CuCOF, CuCOF^+^, CuCOF‐Pd and CuCOF^+^‐Pd, and CO_2_R intermediates of CuCOF^+^‐Pd via ER and LH mechanisms. Energy profiles of CO_2_R to produce CO via ER (D) and LH (E) mechanisms by CuCOF‐Pd and CuCOF^+^‐Pd catalysts.

To deeply understand the CO_2_R mechanism of cationic COFs, four chemical models of CuCOF, CuCOF^+^, CuCOF‐Pd, and CuCOF^+^‐Pd materials were established by carrying out the DFT theoretical calculations. As illustrated in **Figure** **5**A, a Pd cluster with five atoms was used as the model structure of Pd NPs, which is anchored in the pore structure of COFs. Prior to the catalytic mechanism analysis, we calculated the partial density of states (PDOS) of Pd 4d to describe the relationship between the electronic structure of Pd clusters and its CO_2_R performance. As shown in Figure S17 (Supporting Information), the distribution of Pd 4d is mainly below its Fermi level (*E*_F_), and the occupation of electronic states in CuCOF^+^‐Pd presents an upshift to above *E*_F_, indicating that the Pd cluster has a strong interaction with the cationic CuCOF^+^. Since the electron filling depends on the position of the Pd 4d orbital state relative to the *E*_F_ of COFs, the increased binding energy in CuCOF^+^‐Pd indicates that the positively charged CuCOF^+^ induces the charge redistribution of the Pd cluster the partial migration to the COFs.

To unveil the role of the positive charge in supporting the activity of Pd NPs, we analyzed the differences in charge density between CuCOF‐Pd and CuCOF^+^‐Pd. As shown in Table S3 (Supporting Information), the calculated Bader charge shows that a charge redistribution primarily occurs at Pd clusters, in which the electrons accumulate on the epitaxial Pd atoms to form an electron‐rich region. Compared with CuCOF‐Pd, the ethyl‐modified CuCOF^+^ induces the redistribution of electron density on the Pd cluster, which could accumulate efficient electron distribution on the outermost Pd atom, facilitating the adsorption and activation of CO_2_ molecules. This highlights the importance of controlling the charge of the COFs to regulate the electronic properties of supported Pd nanoparticles for improved catalytic activity.

Subsequently, we analyzed the CO_2_ adsorption and activation in the CuCOF and CuCOF^+^ in the presence or absence of Pd clusters. Without Pd NPs, the free energy changes of CO_2_ adsorption by CuCOF and CuCOF^+^ are +0.13 and +0.21 eV, respectively, and the physically adsorbed CO_2_ approaches the C═N group in the pore structure of COFs. In contrast, the free energy changes of CuCOF‐Pd and CuCOF^+^‐Pd for CO_2_ adsorption are −0.87 and −1.36 eV, respectively, and the adsorbed CO_2_ is bonded to the Pd cluster via C─Pd and O─Pd coordination bonds (Figure 5C). These results indicate that the Pd cluster has a significant adsorption and activation capacity for CO_2_ molecules, which can overcome the challenges of CO_2_ adsorption and activation under harsh conditions.

The catalytic conversion process of CO_2_ to CO is usually divided into four steps: CO_2_ adsorption, ^*^COOH and ^*^CO formation involving a proton‐coupled single electron transfer (PCET), and CO desorption. For the CuCOF and CuCOF^+^ catalysts, the free energy change of CO_2_R intermediates from ^*^CO_2_ to ^*^COOH has a large energy barrier of +1.74 and +1.52 eV, respectively, which was identified as the rate‐determining step of CO_2_R (Figures S18 and S19, Supporting Information).^[^ ^15^, ^19^ ^]^ Despite the marginally lower energy for the charged CuCOF^+^ by 0.22 eV compared to the CuCOF, the CO_2_ to CO conversion process remains challenging. This aligns with the low catalytic activities of both COFs in the electrochemical experiments. After loading Pd clusters or nanoparticles on CuCOFs, the adsorption and activation process of CO_2_ occurs on the Pd atom, which is identified as the active site of the CO_2_R reaction. From the perspective of reaction kinetics, we compared the Pd catalyst sites for the conversion pathways from ^*^CO_2_ to ^*^CO under Eley–Rideal (ER) and Langmuir–Hinshelwood (LH) In the ER mechanism, the ^*^CO_2_ intermediate obtains protons and electrons from the solution via a PCET process to produce ^*^COOH intermediate, which is then converted to ^*^CO and leaves a molecule of H_2_O. As illustrated in Figure 5D, the free energy diagram reveals that the formation of ^*^COOH remains the rate‐determining step, but with a notably decreased free energy of 0.93 and 0.59 eV for CuCOF‐Pd and CuCOF^+^‐Pd, respectively. In contrast, the LH mechanism predominantly undergoes intermediates that simultaneously adsorb CO_2_ and hydrogen atoms, thereby facilitating the hydrogenation of ^*^CO_2_ on the surface of Pd NPs. Hence, after CO_2_ and hydrogen adsorption on CuCOF‐Pd and CuCOF^+^‐Pd, the formation of ^*^COOH intermediate overcomes the transition state (TS) with a relatively low barrier energy of 0.71 and 0.46 eV, respectively (Figure 5E). Additionally, the subsequent step for ^*^COOH to ^*^CO conversion requires an energy barrier of ≈0.83 and 0.58 eV, respectively. These results suggest that the conversion of ^*^CO_2_ to ^*^CO on Pd‐supported CuCOF and CuCOF^+^ is more favorable through the LH mechanism than the ER mechanism. Besides, the CuCOF^+^‐Pd catalyst exhibits a facile pathway to convert CO_2_ to CO, aligning with its highest activity of CO_2_R and selectivity of CO products. To explain the selectivity of CO product, the hydrogenation of ^*^CO to form either ^*^COH or ^*^HCO intermediate was investigated, which are the initial steps for the formation of CO‐free products. As illustrated in Figure S20 (Supporting Information), the energy profiles of CO desorption on CuCOF^+^‐Pd was only +0.08 eV. In contrast, the energy consumption of the formation of ^*^COH or ^*^HCO intermediate via the ER mechanism is higher than the CO desorption process, reaching +0.28 and +0.43 eV, respectively. On the other hand, the hydrogenation of CO via the LH mechanism requires a high activation energy barrier of ≈0.91 eV to form ^*^COH intermediate and an even higher barrier of 1.59 eV to form ^*^HCO intermediate. These results indicate that the CO desorption is more favorable than the CO hydrogenation process, ensuring high selectivity of CO products. Hence, introducing a positive charge group and Pd catalyst on the COFs could significantly boost CO_2_ adsorption and activation, enhancing the integrated CO_2_R activity and selectivity.

## 3 Conclusion

In summary, two Cu‐coordinated COFs have been successfully prepared by traditional solvothermal synthesis methods. The two COFs have shown better CO_2_‐capturing abilities and electrocatalytic CO_2_R performances. Upon comparing the catalytic performances of two COFs, CuCOF^+^ decorated with extra ethyl groups exhibits a main CO_2_R product of CO with selectivity of 57.81%, outperforming the CuCOF with 42.92% of CO product at the same applied potential of 0.8 V_RHE_. CO_2_ adsorption and good conductivity, confirmed by gas adsorption and electrochemical analysis. After loading Pd nanoparticles, CuCOF^+^‐Pd has demonstrated the highest selectivity with FE_CO_ of 95.45% higher than that of CuCOF FE_CO_ of 84.97% at −0.8 V_RHE_. However, CuCOFs without Pd NPs have shown less catalytic toward CO_2_R. The positive charge has facilitated the free passage of CO_2_ and hindered water molecules onto the catalytic surface. The highest selectivity of Pd confide CuCOFs to CO is due to the synergistic effects of Pd NPs as depicted by DFT calculations. Therefore, this study provides solid insights into the efficiency and applications of CuCOF materials for CO_2_ capture and CO_2_R.

## Conflict of Interest

The authors declare no conflict of interest.

## Supporting information

Supporting Information

## Data Availability

The data that support the findings of this study are available in the supplementary material of this article.
