# Supplementary material for: Molecularly Woven Cationic Covalent Organic Frameworks for Highly Selective Electrocatalytic Conversion of CO2 to CO
Source: Adv Sci (Weinh). 2024 Sep 10;11(42):2408152. doi: 10.1002/advs.202408152 (PMC11558085; doi:10.1002/advs.202408152)
Supplement: Supplementary file 1 — Supporting Information [file ADVS-11-2408152-s001.docx]

Supporting Information

Molecularly Woven Cationic Covalent Organic Frameworks for Highly Selective Electrocatalytic Conversion of CO_2_ to CO

Fentahun Wondu Dagnaw,^[a],†^ Karim Harrath,^[b],[c],†^ Tao Zheng,^[a]^ Xu-Dong Wu,^[a]^ Yu-Ze Liu,^[a]^ Rui-Qi Li,^[a]^ Luo-Han Xie,^[a]^ Zhen Li,^[a]^ Xuezhong He,^[d]^ Qing-Xiao Tong*^[a]^ and Jing-Xin Jian*^[a]^

[a] Dr. F. W. Dagnaw, T. Zheng, X. D. Wu, Y.-Z. Liu, R.-Q. Li, L.-H. Xie, Dr. Z. Li, Prof. Dr. Q.-X. Tong, Dr. J.-X. Jian, *Department of Chemistry, Key Laboratory for Preparation and Application of Ordered Structural Materials of Guangdong Province, and Guangdong Provincial Key Laboratory of Marine Disaster Prediction and Prevention, Shantou University, Guangdong 515063, P. R. China*

E-mail: *qxtong@stu.edu.cn*; *jxjian@stu.edu.cn*

[b] Dr. K. Harrath, Department of Chemistry, Southern University of Science and Technology, Shenzhen, 518055, China

[c] Dr. K. Harrath, Fundamental Science Center of Rare Earths, Ganjian Innovation Academy of Sciences, Ganzhou 431000, China

[d] Prof. X. He, Department of Chemical Engineering, Guangdong Technion - Israel Institute of Technology, Shantou 515063, China

^†^ F. W. D. and K. H. contributed equally.

**1. Experimental**

**1.1 Chemicals and Materials**

The chemicals and reagents such as 3, 8-Diamino-5-ethyl-6-phenylphenanthridin-5-ium bromide, Cu(CH_3_CN)BF_4_, 4,4'-(1,10-phenanthroline-2,9-diyl)dibenzaldehyde, and 6-phenylphenanthridine-3,8-diamine were purchased from Bidepharm Chemical company. Unless otherwise stated, all reagents and solvents used for the synthesis of Cu-COFs and starting materials were used as received without any further purification. Moisture-sensitive reactions were performed in an oven-dried round bottom flask under a dry nitrogen atmosphere. Bruker AV spectrometer operating at 400 was measured on ^1^H NMR spectra of reference compounds. Internal standard tetramethylsilane (TMS, σ = 0.00 ppm) was used to record chemical shifts of compounds. Moreover, CDCl_3_ and DMSO-*d6* with their peaks at 7.26 and 2.50 ppm for ^1^H NMR spectra respectively were applied to determine the chemical shifts of the reference compounds. Solid-state UV-Vis spectrometry was performed on UV-8000 Instrument. Surface potential, Zeta potential, and contact angle characterizations of the Cu-COFs with and without Pd nanoparticles were performed by Kelvin Probe Force Microscopy (KPFM), Zetasizer Nano ZS90, and Biolin Scientific respectively.

**1.2 Synthesis of [Cu(PDB)_2_]BF_4_**

**Scheme S1.** Synthesis of [Cu(PDB)_2_]BF_4_.

The preparation of Cu(PBD)_2_]BF_4_ was done according to the literature.^[1]^ Hence, the solution of Cu(CH_3_CN)BF_4_ (0.0831 g, 0.2643 mmol) in acetonitrile (3 mL) was added to a solution of 4,4'-(1,10-phenanthroline-2,9-diyl)dibenzaldehyde (0.2053 g, 0.5286 mmol) in DCM (13 mL) dropwise The dark red reaction mixture was then stirred for 3 h at room temperature. After the completion of the reaction, the solvent was removed under reduced pressure. The residue was recrystallized with DCM and PE (1:1, V/V) to get a dark red solid product. Finally, the product was characterized by HR-MS (**Figure S1**), ^1^H-NMR (**Figure S2**) and FT-IR (**Figure S3**).


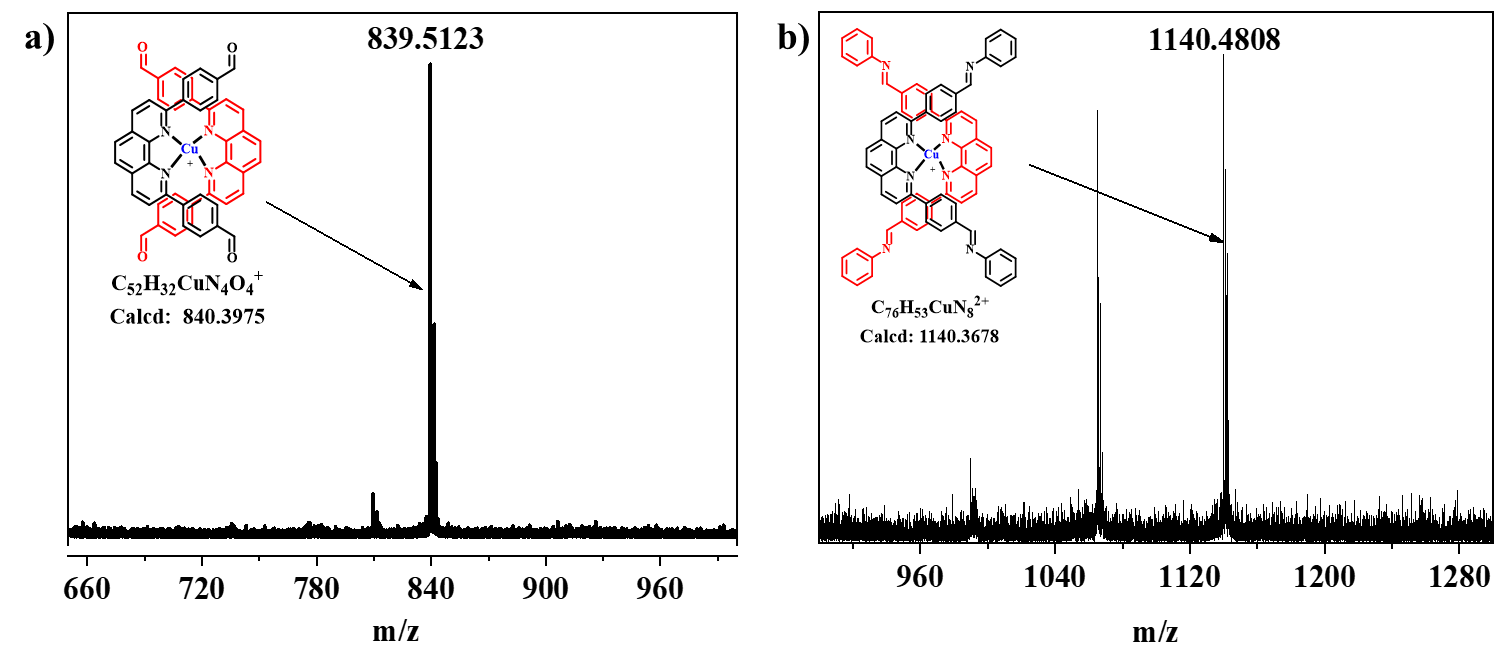


**Figure S1.** HR-MS spectra of Cu(PDB)_2_.


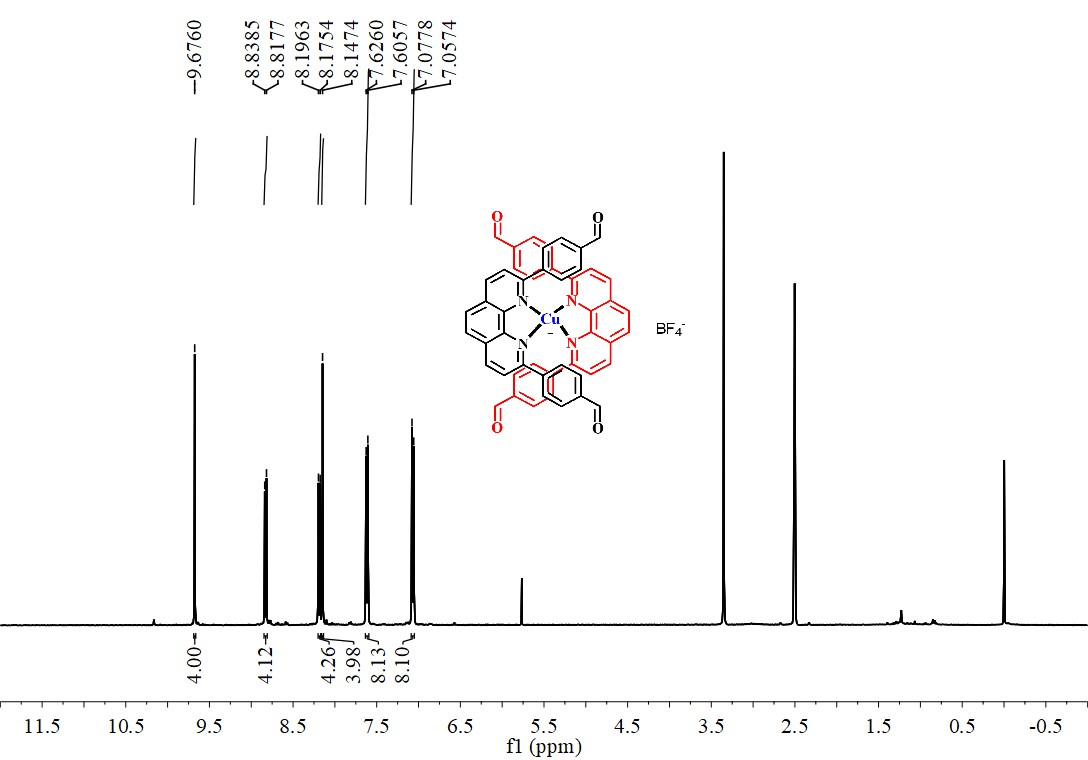


**Figure S2.** ^1^H-NMR spectra of Cu(PDB)_2_BF_4_.

**1.3 Synthesis** **of CuCOF**

**Scheme S2.** Synthetic route for CuCOF.

CuCOF was synthesized according to the literature by modification of experimental conditions and procedures. Accordingly, the mixture of Cu(PDB)_2_BF_4_ (0.0379 g, 0.0410 mmol), 6-phenylphenanthridine-3,8-diamine (0.0233g, 0.0820 mmol) and aqueous solution of acetic acid (0.2 mL, 6M) in 4 mL dimethylforamide and chlorobenzene (7:3, v/v) was freeze-pump-thawed three times. The reaction mixture was then heated in a sealed tube at 140 ºC under gentle stirring for 5 days. After cooling to r.t, it was filtered and washed several times with DCM, THF, and 1,4-dioxane to remove unreacted starting materials and dried in the oven at 80 ºC overnight. Finally, the successful prepared CuCOF was characterized by FT-IR, PXRD, XPS, and TEM.

**1.4 Synthesis** **of CuCOF^+^**

**Scheme S3.** Synthetic route for CuCOF^+^

Synthesis of CuCOF^+^ was performed in the same fashion as that of CuCOF except changing the amine precursor to positively charged pyridine moiety. To do so, the mixture of Cu(PDB)_2_BF_4_ (0.0383 g, 0.0413 mmol), 3, 8-Diamino-5-ethyl-6-phenylphenanthridin-5-ium bromide (0.0326g, 0.0826 mmol) and aqueous solution of acetic acid (0.2 mL, 6M) in 4 mL 1:1 mixture of 1,4-dioxane and mesitylene was freeze-pump-thawed three times. The reaction mixture was then heated in a sealed tube at 120 ºC under gentle stirring for 7 days. After cooling to r.t, it was filtered and washed several times with DCM, THF, and 1,4-dioxane to remove unreacted starting materials and dried in the oven at 80 ºC overnight. Finally, the successful prepared CuCOF^+^ was characterized by FT-IR, PXRD, XPS, and TEM.

**Figure S3**. FT-IR spectra of PhDA, EB, Cu(PDB)_2_BF_4_, CuCOF and CuCOF^+^.


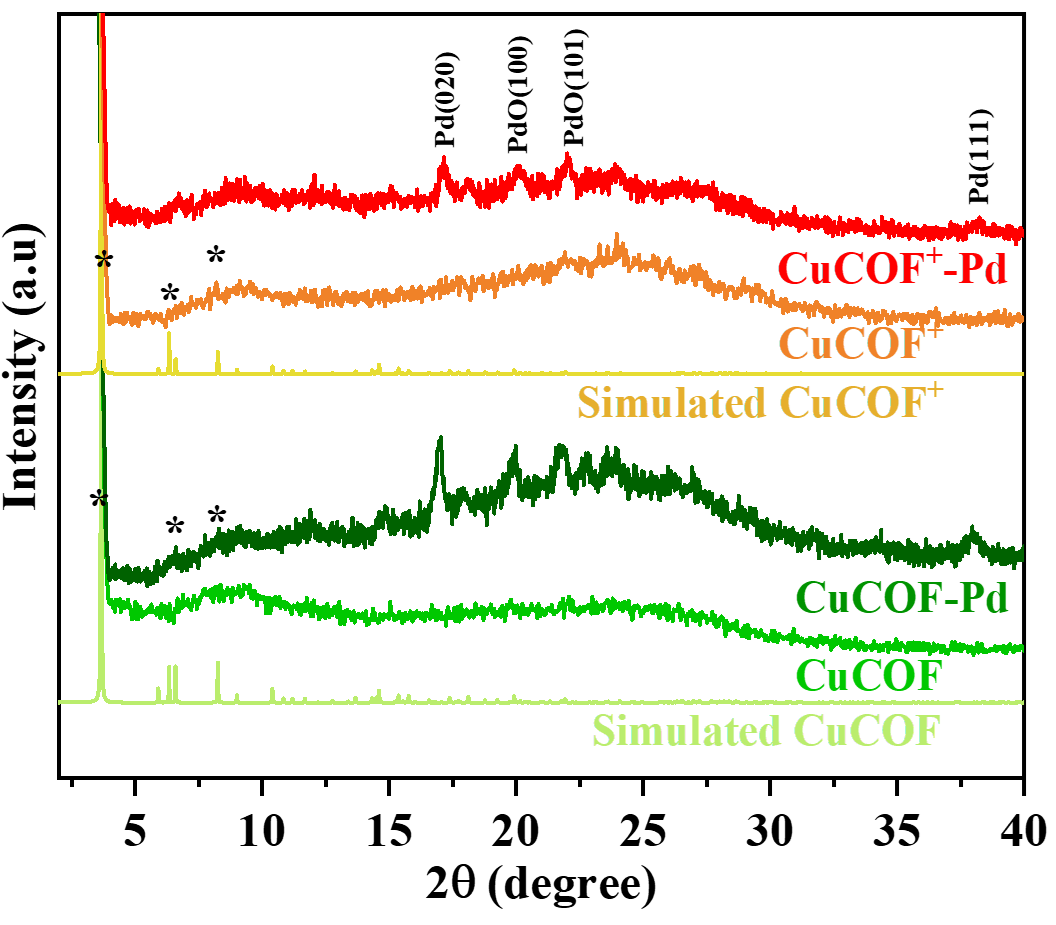


**Figure S4.** PXRD spectra and the simulated results of CuCOF, CuCOF-Pd, CuCOF^+^ and (b) CuCOF^+^-Pd, respectively.


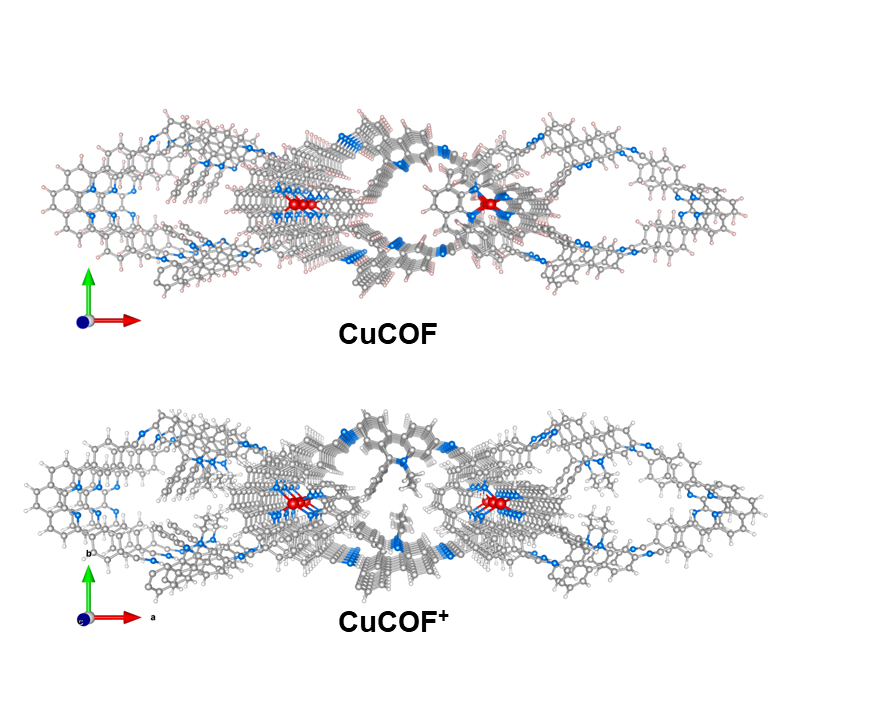


**Figure S5.** Structural models of CuCOF and CuCOF^+^.

**1.5 Electrochemical measurements**

1. **Activation of Carbon Cloth electrode**

Before the preparation of the working electrode, the carbon cloth electrode was cleaned in distilled water and ethanol three times for 30 minutes. Then, it was inserted into Teflon-lined stainless steel containing a mixture of distilled water and HNO_3_ (2:1, V/V) and heated at 140 ºC for 210 minutes. Finally, it was washed with distilled water and dried in the oven at 60 ºC.

1. **Preparation of working electrode**

The working electrode was prepared by cutting about 1.5 cm^2^ of a pre-treated carbon cloth electrode. Then, part of the CC electrode was covered by glue to protect the movement of the electrolyte solution into Pt foil and avoid false current readings. It was then dried overnight at room temperature. The working electrode (WE) was then prepared by drop casting the sonicated solution of the mixture of Cu-COF materials on the surface of the pretreated CC electrode. Accordingly, the mixture of 3.0 mg of Cu-COFs, 15 µL Nafion solution, and 1.0 mg Pd nanoparticle in 0.3 mL DMF was sonicated until it was fully dissolved. 40 µL of dissolved ink (around 1mg/mL) was then drop-casted onto the surface of the carbon cloth electrode and heated at 50 ºC until the solvent was fully evaporated and the electrode was dried enough.

1. **Electrochemical measurement**

All electrochemical measurements were conducted in a three-electrode electrolytic cell system consisting of a carbon cloth electrode as a working electrode (WE), Pt sheet electrode as a counter electrode (CE), and Ag/AgCl (saturated KCl) as reference electrodes (RE) by electrochemical workstation (CHI-760). Before EC-CO_2_RR measurement, the electrolyte solution containing 0.1 M KHCO_3_ was bubbled with high-purity nitrogen (99.999%) and CO_2_ (99.999%) for 30 minutes separately. The LSV curves were recorded within the potential windows of -0.6 to -2 V at a scanning rate of 50 mV/s. All reference electrodes are converted to RHE scale by E(*vs.* RHE) = **E** (***vs****.***Ag/AgCl)** +**0.197 V + 0.0591 V** × pH, where the pH of CO_2_ saturated 0.1 M KHCO_3_ solution was determined to be 6.8.

The gaseous products under the EC-CO_2_RR system on Cu-COFs and Cu-COFs-Pd deposited on carbon cloth electrodes were CO, CH_4_, and H_2_. The amount of generated gaseous products was quantified by online gas chromatography (GC) analysis (GC-7920). The FE of the photoelectrochemical reduction of CO_2_ process was calculated as follows, by the corresponding peak area present in the chromatogram, which is calibrated by a certified standard gas.

FE = (Q_output_ / Q_input_) × 100% = I_p_/ I_t_ × 100%

I_output_ = V_p_ × P × nF/RT

V_p_ = α_p_ × V_t_

Where, α_p_ is the volume fraction of the product derived from GC calibration, V_t_ is the total gas flow rate (sccm), and R, T, P, n, F, I_p_ and I_t_ represent the ideal gas constant, temperature, pressure, the number of electrons transferred to the product formation during CO_2_ reduction, faradic constant, the current of product calculated from GC data and the total current respectively.

**1.5 *In Situ* ATR-FTIR Measurements**

The electrocatalytic CO_2_R reactive intermediates on the CuCOF-Pd and CuCOF^+^-Pd were carried out by *in situ* ATR-SEIRAS. To do so, the solution of Cu-COFs-Pd in ethanol was dropped on the surface silicon electrode coated on Au. It was then dried at room temperature and attached to the catalytic cell. Finally, CO_2_ saturated 0.1 M KHCO_3_ electrolyte solution was added to the cell and a bias potential was applied from the electrochemical workstation (CHI-660E) with continuous babbling of CO_2_ into the solution followed by detecting the intermediates at different applied potentials. Taking the test results under-0.4 V_RHE_ as the baseline, the change of infrared signal peak after voltage change is observed more clearly.

- 1. **DFT Calculation**

The spin-polarized DFT calculations with the Perdew-Burke-Ernzerhof (PBE) exchange-correlation functional ^[2]^ were performed using the Vienna ab initio simulation package (VASP).^2^ The projector augmented wave method (PAW)^[3]^ with a plane-wave kinetic energy cutoff of 500 eV with the Gaussian smearing of 0.05 eV was used. The Brillouin zone was sampled by 3×1×3 K-point for geometry optimization and 6×1×6 K-point to calculate the PDOS.^[4]^ The model of the CuCOF material was constructed based on experiment results with a lattice constant of a= 17.966 Å, b= 33.094 Å, and c= 7.139 Å, as shown in Figure S5. Pd NPs confined COF model was constructed with a small Pd_5_ cluster on CuCOF to minimize the calculation time during CO_2_ adsorption and CO desorption All atoms were allowed to relax during geometry optimization, and the atomic positions were optimized till the forces were less than 0.03 eV/Å. The lattice dimensions were optimized simultaneously with the geometry. The structure of isolated molecules (O_2_, H_2_, and H_2_O) was optimized within a unit cell measuring 15Å×15 Å×15 Å, with only the Γ-point utilized. ^[5]^ The effects of van der Waals corrections were modeled using Grimme's method with Becke-Jonson damping. ^[6]^ The transition states (TS) were searched by the Dimer method^[7]^ and further confirmed by vibrational frequency analysis. Only one imaginary frequency was found for each TS structure reported in this work.

The binding energy of the CO_2_ is calculated based on the following equation:

$E_{binding}^{CO2} = E_{total} - E_{surafce} - E_{CO2})$, where$E_{surafce}$ is the energy of the CuCOFs model with and without the Pd cluster.

The Gibbs free energy of a species is calculated using the following equation:

G = E + ZPE – TS

Where, E is the total energy of a species obtained from DFT calculations, ZPE and S are the zero-point energy and entropy of a species, respectively, and T= 298.15 K.

**Table S1**. BET surface area of CuCOFs in N_2_ adsorption-desorption measurements at 77 K.

| Samples | BET surface area (m^2^/g) |
| --- | --- |
| CuCOF | 38.6079 ± 1.0553 |
| CuCOF^+^ | *2*7.6426 ± 0.*8*744 |
| CuCOF-Pd | 7.0386 ± 0.2025 |
| CuCOF^+^-Pd | 4.0780 ± 0.0064 |


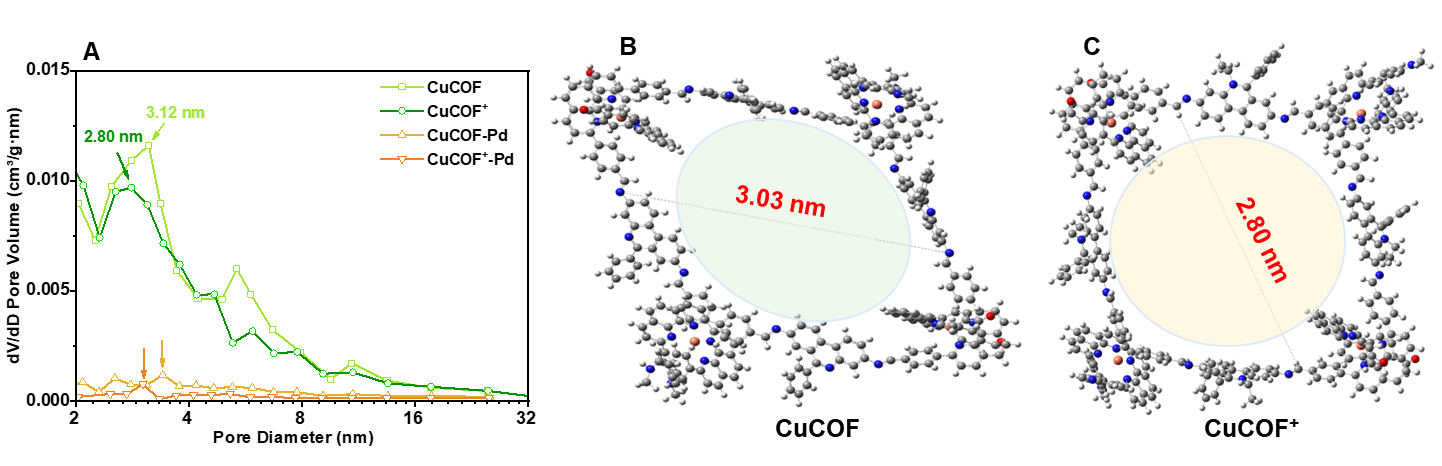


**Figure S6**. (a) BJH desorption pore size distributions of CuCOF and CuCOF^+^, CuCOF-Pd and CuCOF^+^-Pd in N_2_ adsorption-desorption measurements at 77 K. Theoretically calculated pore sizes of CuCOF (B) and CuCOF^+^ (C) in the optimized DFT models.


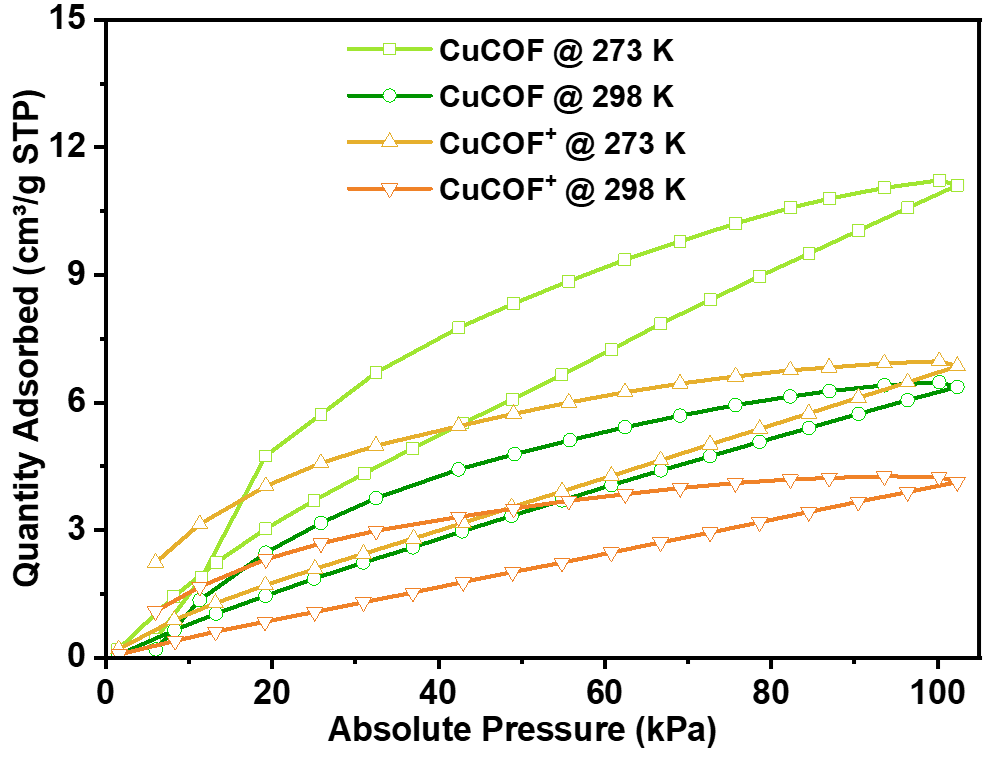


**Figure S7.** CO_2_ adsorption-desorption measurements of CuCOF and CuCOF^+^ at 273 and 298 K.


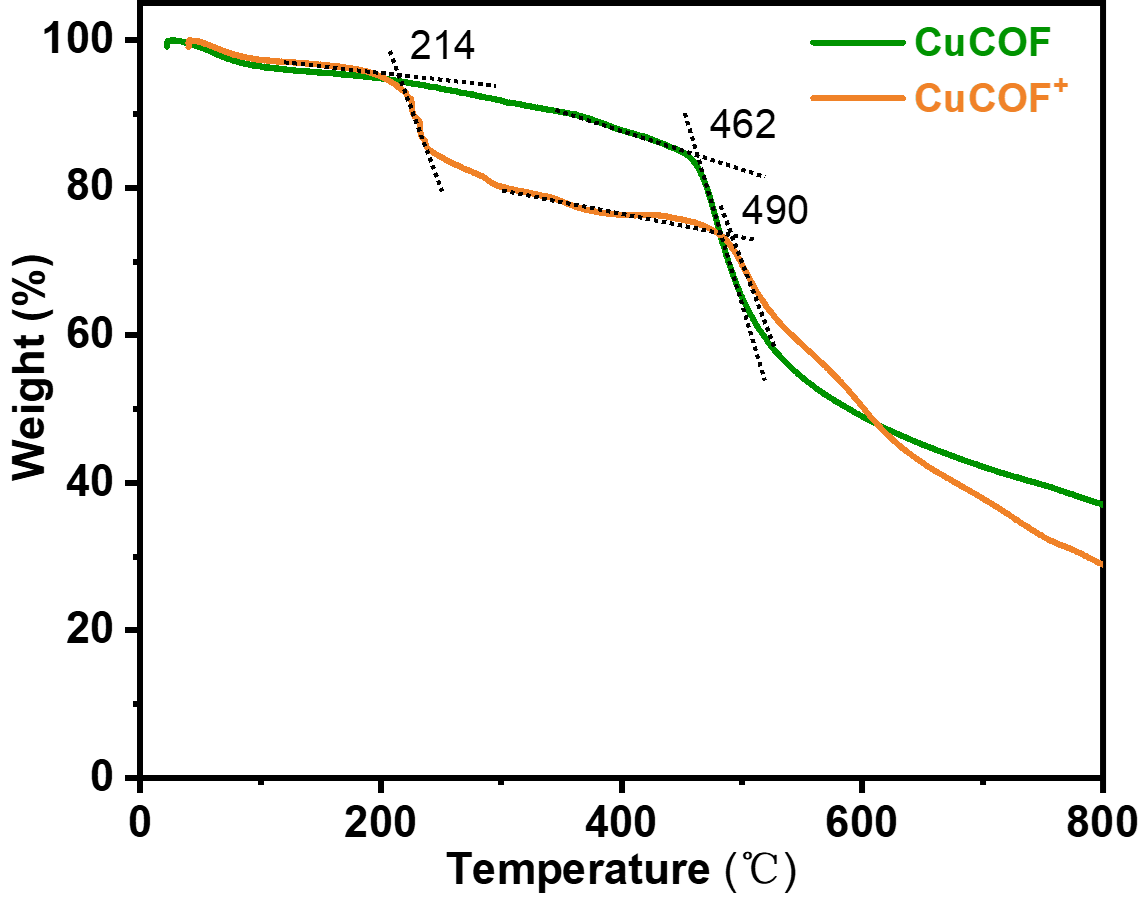


**Figure S8.** TGA analysis of CuCOF and CuCOF^+^.


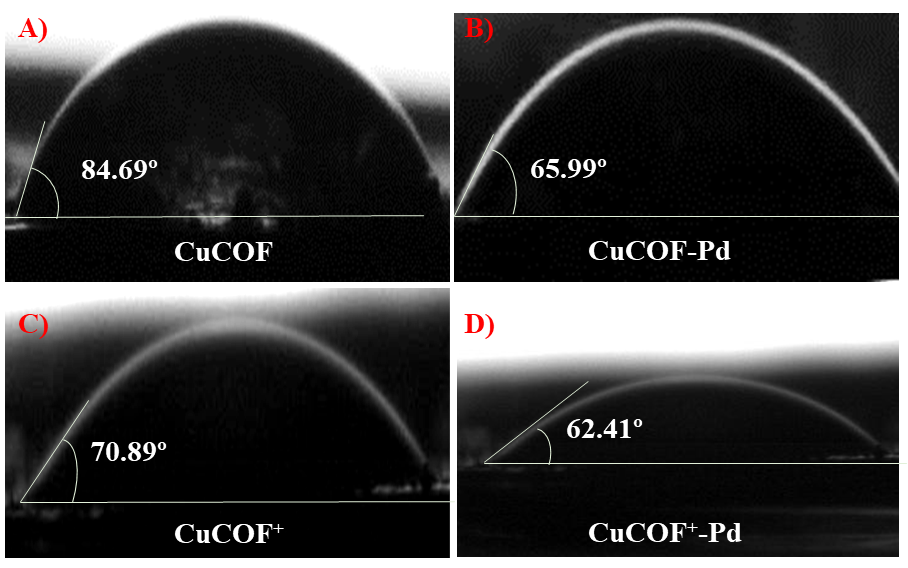


**Figure S9**. Contact angles (A CuCOF, B: CuCOF-Pd, C: CuCOF^+,^ and D: CuCOF^+^-Pd) respectively.


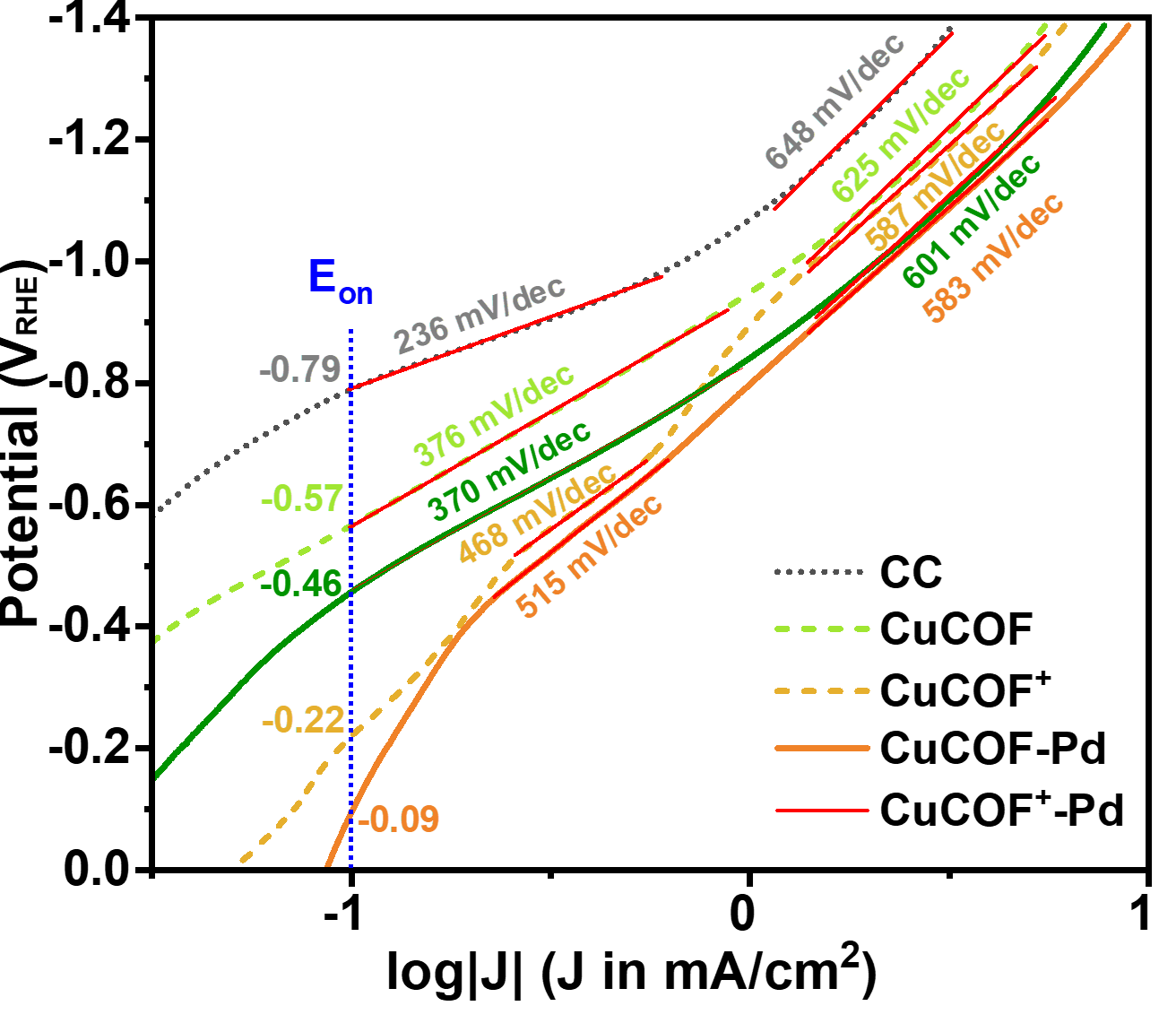


**Figure S10**. Tafel plots of CC, CuCOF, and CuCOF^+^ with and without Pd NPs.

**Table S2.** Summary of synergistic catalysts for CO_2_R to CO

| No | Catalyst | Electrolyte | Potentials (V_RHE_) | FE(%) | Ref. |
| --- | --- | --- | --- | --- | --- |
| 1 | N-C-CoPc NR | 0.1 M KHCO_3_ | -0.7 | 85.3 | **^[8]^** |
| 2 | [Ni_2_L**^1^**](ClO_4_)_4_ | 0.1 M TBAPF_6_ | -1.16 V_NHE_ | 95 | **^[9]^** |
| 3 | CuO/In_2_O_3_ | 0.1 M KHCO_3_ | -0.7 | 93 | **^[10]^** |
| 4 | Zn-N-G | 0.5 M KHCO_3_ | 0.39 | 91 | **^[11]^** |
| 5 | PcCu-O8-Zn/CNT | 0.1 M KHCO_3_ | -1.0 | 88 | **^[12]^** |
| 6 | TeN_2_-CuN_3_ | 0.1 M KHCO_3_ | -0.65 | 98 | **^[13]^** |
| 7 | FeCo-PPc | 1 K KOH | -0.8 | 90 | **^[14]^** |
| 8 | NiPc/KB | 0.5 M KHCO_3_ | 0.75 | 98.2 | **^[15]^** |
| 9 | NiN_3_©CoN_3_-NC | 0.1 M KHCO_3_ | -1.1 | 97.7 | **^[16]^** |
| 10 | Co–Ni–N–C | 0.1 M KHCO_3_ | -0.27 | 94.1 | **^[17]^** |
| 11 | BPB-Pd2 | 0.5 M KHCO_3_ | -0.8 | 94.4 | **^[18]^** |
| 12 | Pd-Zn NSs | 0.1 M CsHCO_3_ | -0.8 | 88.5 | **^[19]^** |
| 13 | NiFe-DAC | 0.1 M KHCO_3_ | -0.33 | 99.8 | **^[20]^** |
| 14 | NiPc-CoPor-imi-COF | 0.5 M KHCO_3_ | -1.4 V_Ag/AgCl_ | 97.1 | **^[21]^** |
| 15 | D-Co/CNT | 0.5 M KHCO_3_ | -0.90 | 91.76 | **^[22]^** |
| 16 | Ni_SAsNPs_-N-HCSs | 0.5 M KHCO_3_ | - 0.76 | 93.33 | **^[23]^** |
| 17 | PcNi-Co−O | 0.5 M KHCO_3_ | -1.7 | 96 | **^[24]^** |
| 18 | CuCOF-Pd | 0.1 M KHCO_3_ | -0.79 | 84.97 | **This**  **work** |
|  | CuCOF^+^-Pd |  |  | 95.45 |  |


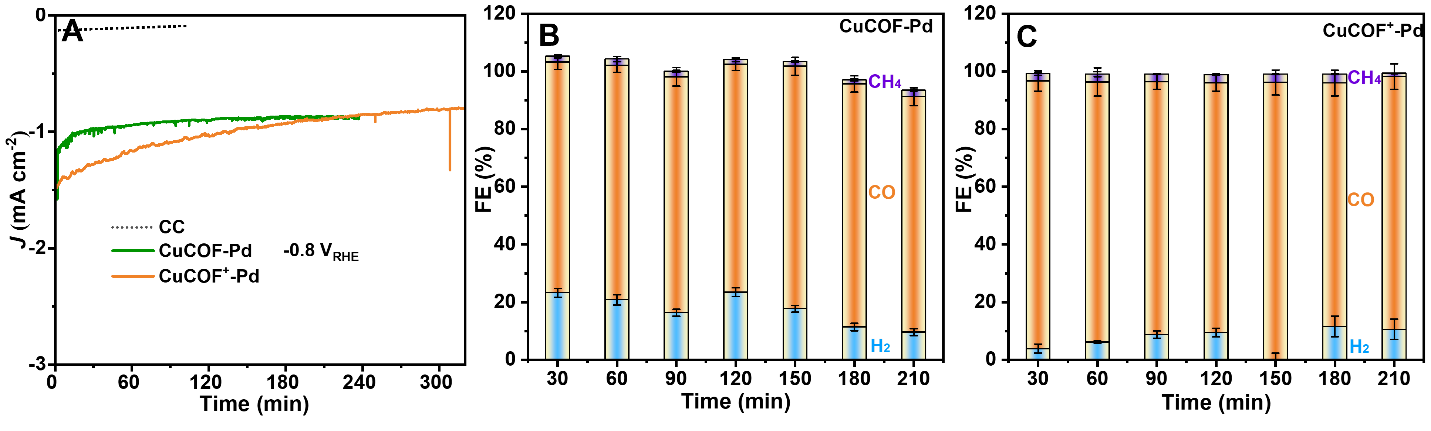


**Figure S11.** I-t curves (A) of CC, CuCOF-Pd, and CuCOF^+^-Pd at -0.8 V_RHE_ and the related FEs of CuCOF-Pd (B) and CuCOF^+^-Pd (C).


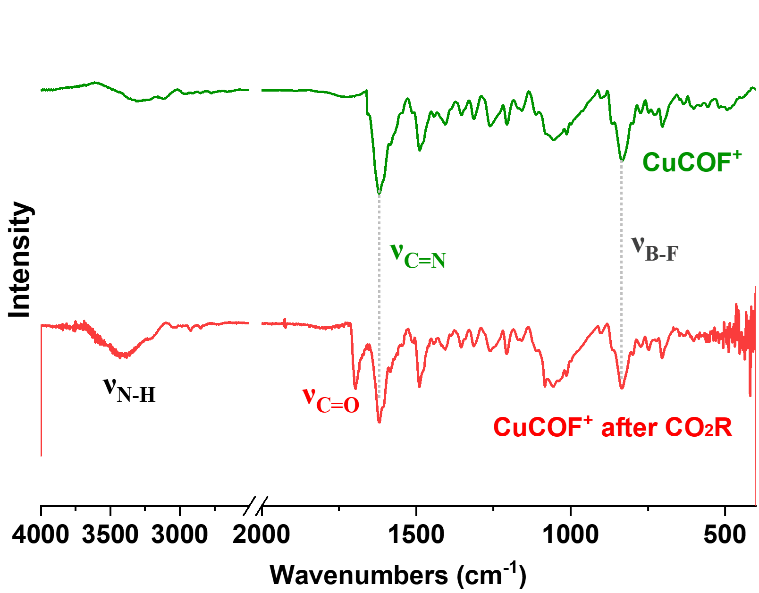


**Figure S12**. FTIR spectra of CuCOF^+^ before and after CO_2_R stability test.


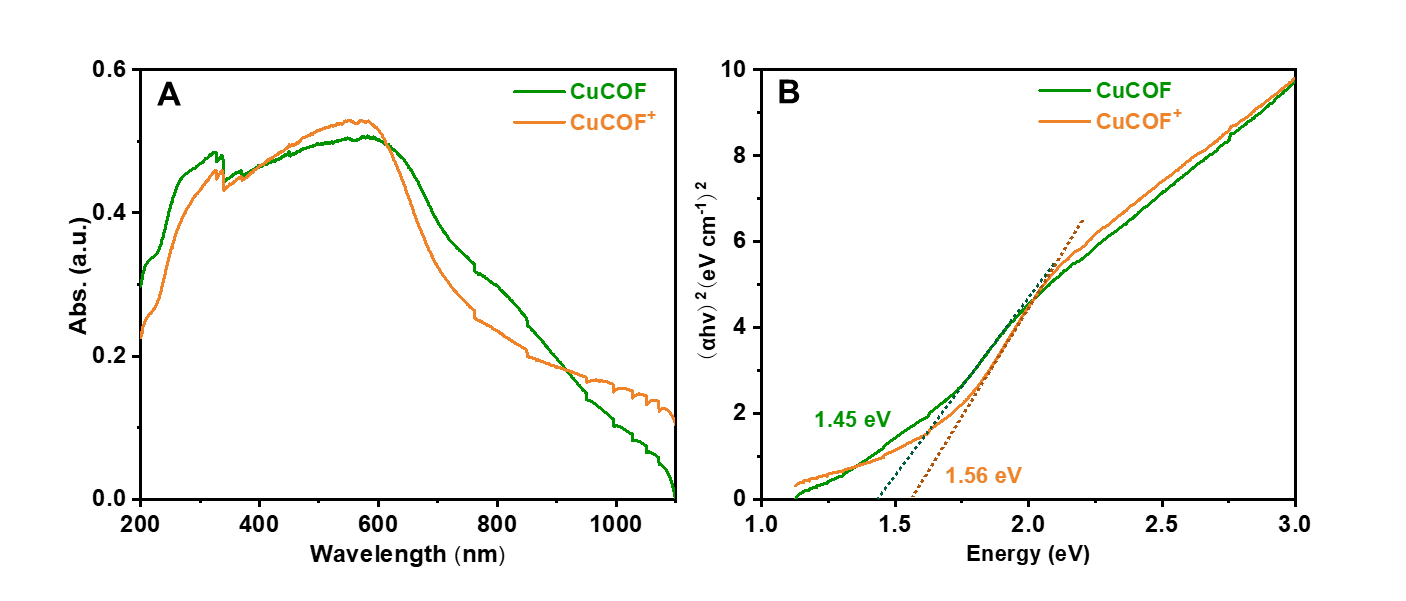


**Figure S13.** Solid state UV-Vis absorption spectra (A) and Tauc plots (B) of CuCOF and CuCOF^+^.

**Figure S14.** Zeta potential analysis of CuCOF, CuCOF-Pd, CuCOF^+^ and CuCOF^+^-Pd.


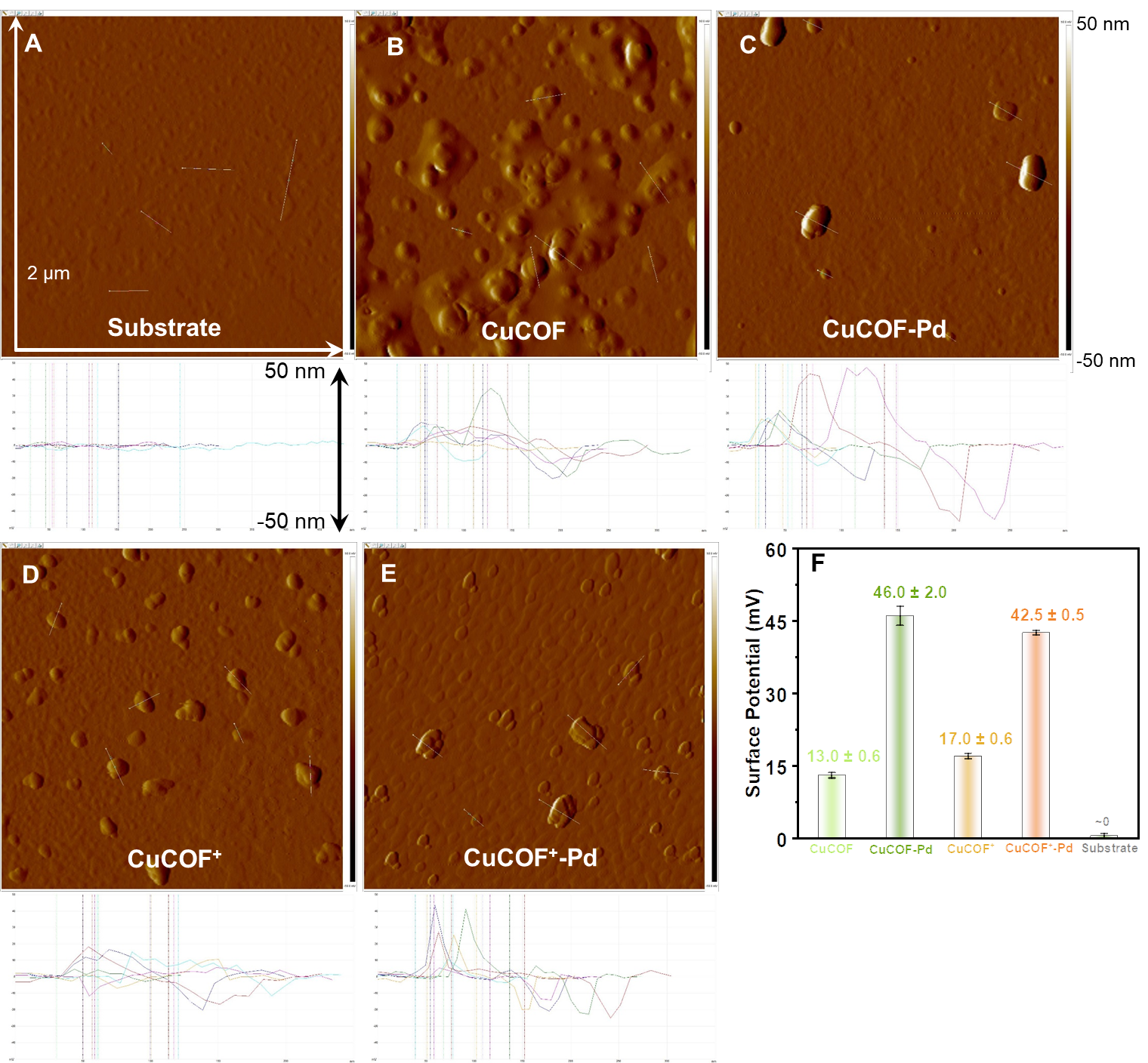


**Figure S15.** Kelvin probe force microscopy (KPFM) images of the substrate (**A**), CuCOF (**B**), CuCOF-Pd (**C**), CuCOF^+^ (**D**), CuCOF^+^-Pd (**E**) and bar graph of surface potential vs Catalyst (**F**).


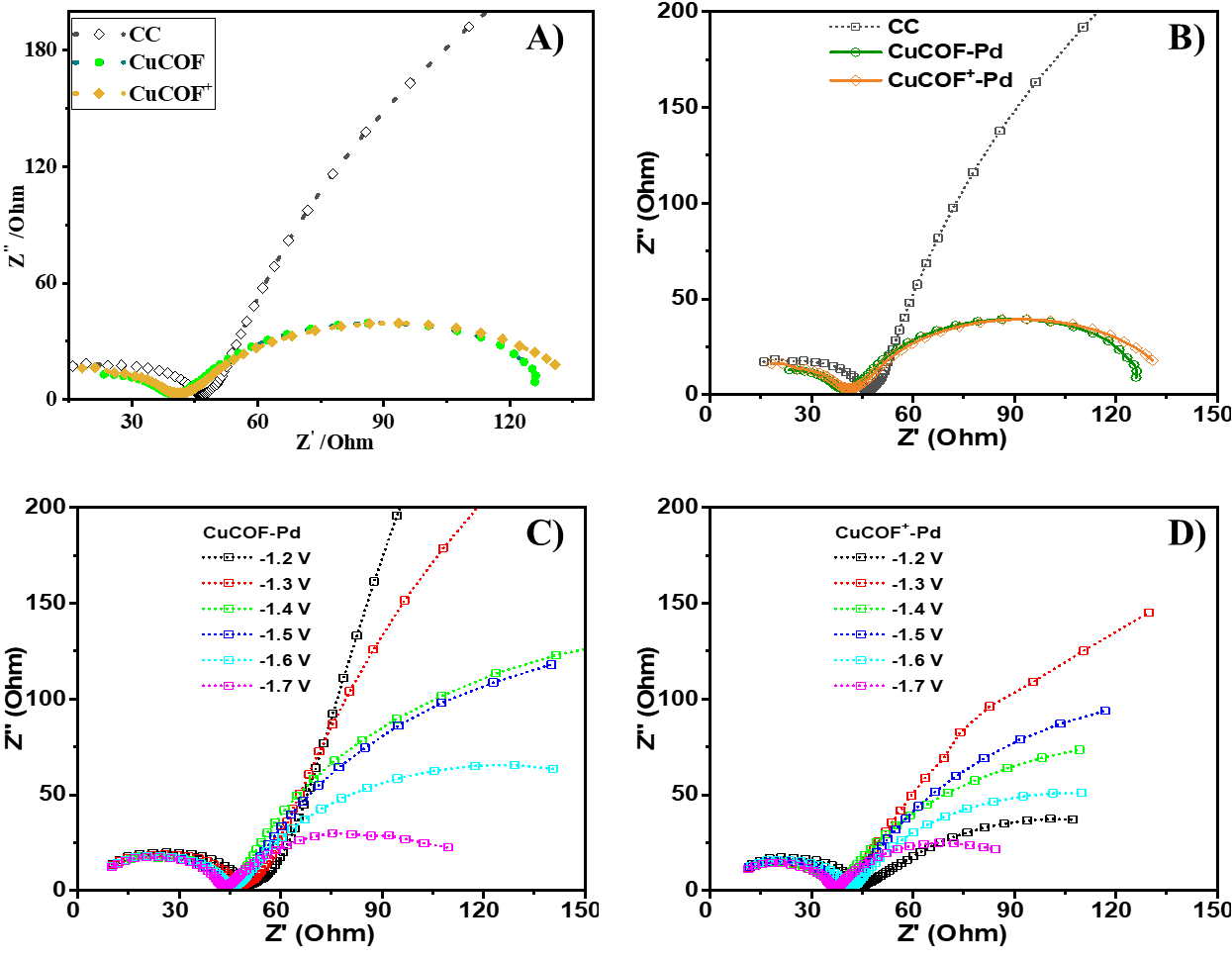


**Figure S16.** EIS of CuCOFs (A) and CuCOFs-Pd (B) at -0.8 V_RHE_, and EIS of CuCOF-Pd (C) and CuCOF^+^-Pd (D) at different potentials (vs. Ag/AgCl).


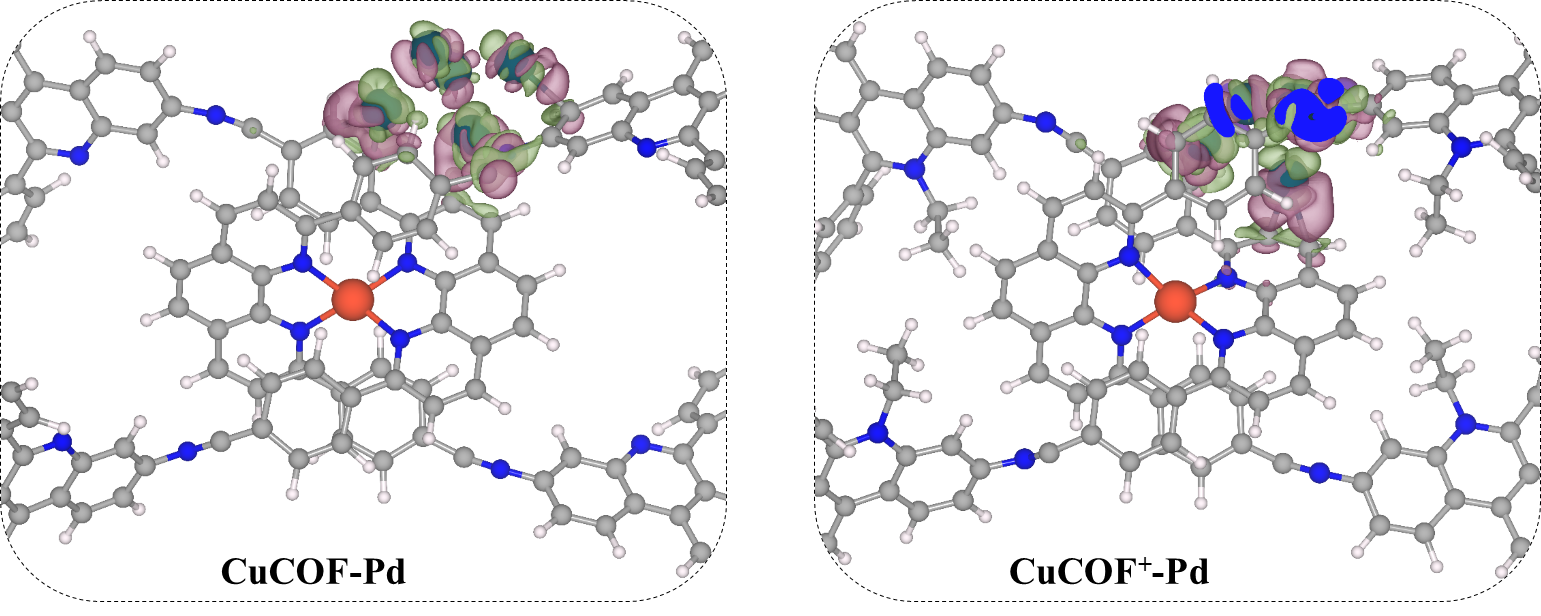


**Figure S17.** Pd cluster charge differences on CuCOF-Pd and CuCOF^+^-Pd.

**Table S3.** Bader charge for Pd atoms from the Pd cluster on CuCOF-Pd and CuCOF^+^-Pd.

| Catalyst | Pd_1_ | Pd_2_ | Pd_3_ | Pd_4_ | Pd_5_ |
| --- | --- | --- | --- | --- | --- |
| CuCOF-Pd | -0.355 | 0.221 | 0.197 | 0.378 | 0.105 |
| CuCOF^+^-Pd | -0.884 | 0.268 | 0.323 | 0.268 | 0.299 |


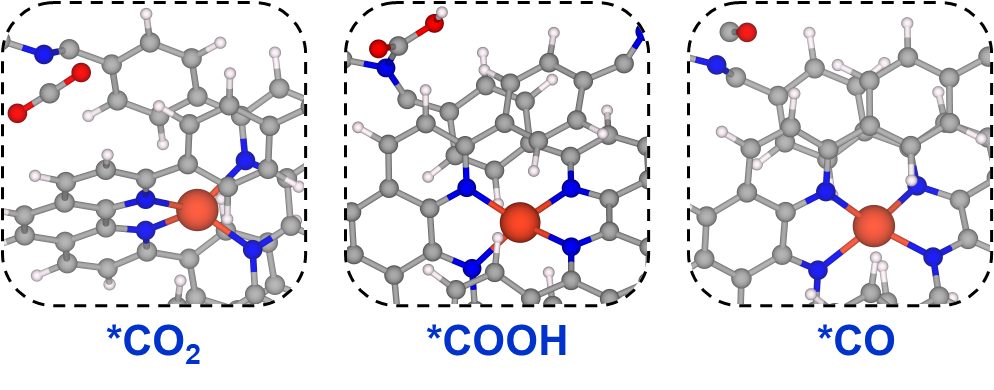


**Figure S18**. The CO_2_R intermediates of CuCOF^+^ *via* ER mechanism.


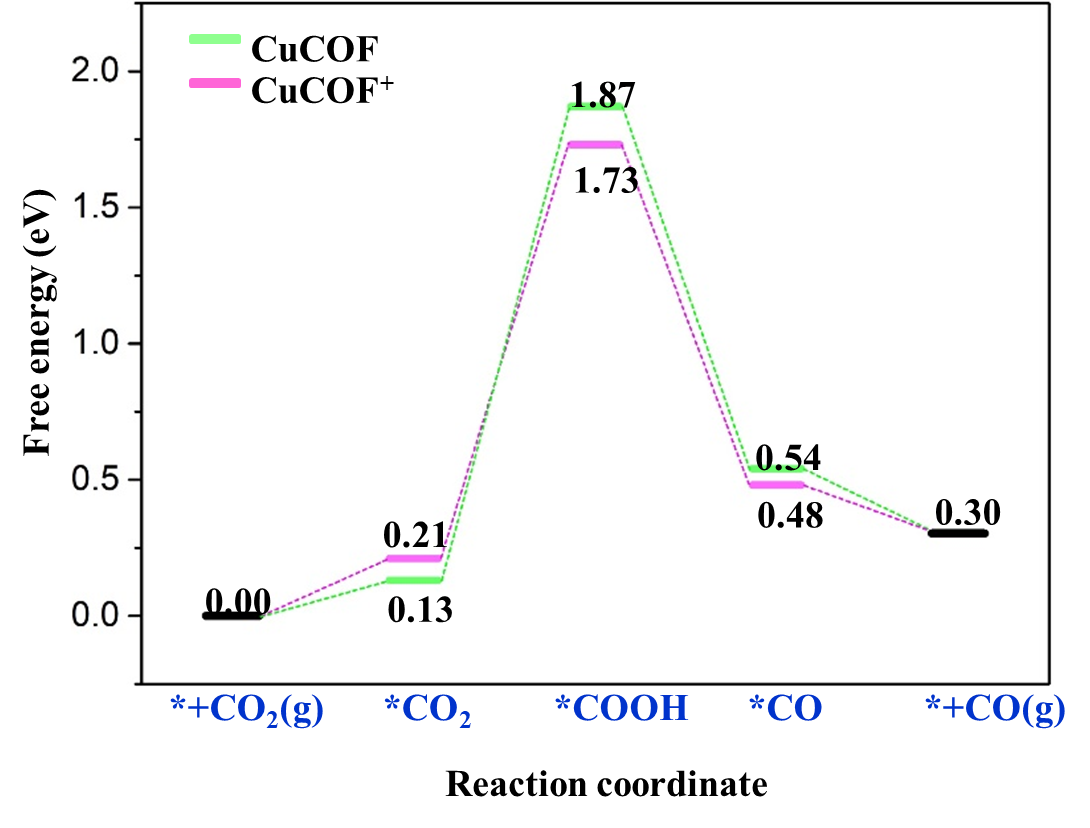


**Figure S19.** Energy profiles of CO_2_R to produce CO via ER by CuCOF and CuCOF^+^.


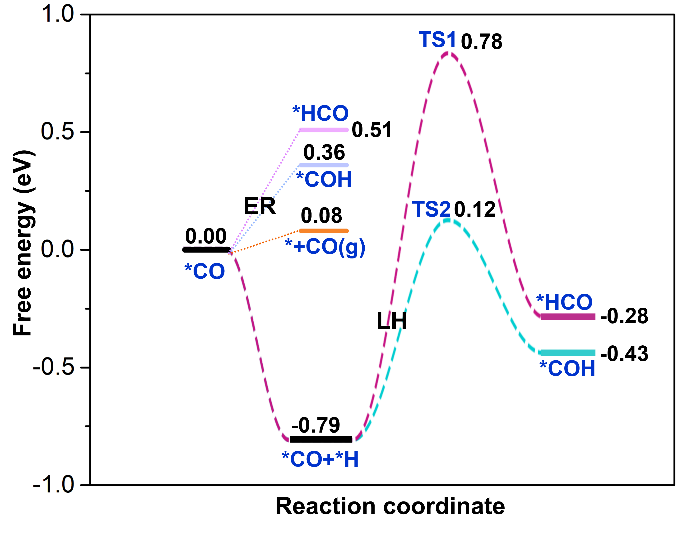


**Figure S20.** Energy profiles of ^*^CO hydrogenation to form ^*^COH or ^*^HCO intermediate *via* ER or LH mechanisms.

**References**

[1] Y. Z. Liu, Y. H. Ma, Y. B. Zhao, X. X. Sun, F. Gandara, H. Furukawa, Z. Liu, H. Y. Zhu, C. H. Zhu, K. Suenaga, P. Oleynikov, A. S. Alshammari, X. Zhang, O. Terasaki, O. M. Yaghi, *Science* **2016**, *351*, 365-369.

[2] J. P. Perdew, K. Burke, M. Ernzerhof, *Phys. Rev. Lett.* **1996**, *77*, 3865-3868.

[3] a) G. Kresse, D. Joubert, *Phys. Rev. B* **1999**, *59*, 1758-1775; b) P. E. Blöchl, *Phys. Rev. B* **1994**, *50*, 17953-17979.

[4] K. Harrath, Z. Yao, Y. F. Jiang, Y. G. Wang, J. Li, *J. Phys. Chem. C* **2024**, *128*, 5579-5589.

[5] a) L. Bahri, F. Mbarki, K. Harrath, *Chem. Pap.* **2023**, *77*, 3759-3767; b) K. Harrath, Z. Yao, Y. F. Jiang, Y. G. Wang, J. Li, *J. Phys. Chem. Lett.* **2023**, *14*, 4033-4041.

[6] a) S. Grimme, J. Antony, S. Ehrlich, H. Krieg, *J. Chem. Phys.* **2010**, *132*, 154104; b) S. Grimme, *Wires Comput Mol Sci* **2011**, *1*, 211-228.

[7] G. Henkelman, H. Jónsson, *J. Chem. Phys.* **1999**, *111*, 7010-7022.

[8] H. L. Zhu, Y. Q. Zheng, M. Shui, *ACS Appl. Energy Mater.* **2020**, *3*, 3893-3901.

[9] L. M. Cao, H. H. Huang, J. W. Wang, D. C. Zhong, T. B. Lu, *Green Chem.* **2018**, *20*, 798-803.

[10] S. L. Chu, S. Hong, J. Masa, X. Li, Z. Y. Sun, *Chem. Commun.* **2019**, *55*, 12380-12383.

[11] Z. Chen, K. Mou, S. Yao, L. Liu, *Chemsuschem* **2018**, *11*, 2944-2952.

[12] H. Zhong, M. Ghorbani-Asl, K. H. Ly, J. Zhang, J. Ge, M. Wang, Z. Liao, D. Makarov, E. Zschech, E. Brunner, I. M. Weidinger, J. Zhang, A. V. Krasheninnikov, S. Kaskel, R. Dong, X. Feng, *Nat. Commun.* **2020**, *11*.

[13] J. Q. Jiao, Q. Yuan, M. J. Tan, X. Q. Han, M. B. Gao, C. Zhang, X. Yang, Z. L. Shi, Y. B. Ma, H. Xiao, J. W. Zhang, T. B. Lu, *Nat. Commun.* **2023**, *14*.

[14] Z. X. Wang, J. Qian, P. C. Cao, H. W. Shou, C. Q. Wu, X. Xu, X. J. Wu, Q. He, L. Song, *Nano Lett.* **2024**, *24*, 3249-3256.

[15] Z. J. Ma, X. L. Zhang, X. Y. Han, D. P. Wu, H. J. Wang, Z. Y. Gao, F. Xu, K. Jiang, *Appl. Surf. Sci.* **2021**, *538*, 148134.

[16] Q. C. Zhang, D. Liu, Y. P. Zhang, Z. L. Guo, M. P. Chen, Y. Y. Chen, B. Jin, Y. Z. Song, H. Pan, *J. Energy Chem.* **2023**, *87*, 509-517.

[17] J. Chen, M. R. Ahasan, J.-S. Oh, J. A. Tan, S. Hennessey, M. M. Kaid, H. M. El-Kaderi, L. Zhou, K. U. Lao, R. Wang, W.-N. Wang, *J. Mater. Chem. A* **2024**, *12*, 4601-4609.

[18] W. X. Zhang, M. R. Zhang, H. J. Wang, W. Zhang, M. Zhang, *Nano Res.* **2024**.

[19] S. A. Mahyoub, F. A. Qaraah, S. L. Yan, Z. H. Yamani, Q. A. Drmosh, A. Hezam, T. N. Baroud, S. A. Onaizi, Z. M. Cheng, *Chemcatchem* **2024**, *16*, e202301517.

[20] H. Han, S. Lee, J. Im, M. Lee, T. Lee, S. T. Hyun, J. Hong, T. Seok, D. Choo, *Chem. Eng. J.* **2024**, *479*, 147603.

[21] X.-Y. Dong, F.-Q. Yan, Q.-Y. Wang, P.-F. Feng, R.-Y. Zou, S. Wang, S.-Q. Zang, *J. Mater. Chem. A* **2023**, *11*, 15732-15738.

[22] L. M. Hong, X. Liu, B. Z. Chi, G. M. Xia, H. M. Wang, *J. Mater. Chem. A* **2023**, *11*, 6321-6328.

[23] M. Li, Y. Hu, W. Fang, S. Xin, Y. Wu, Y. Cao, W. Cui, Z. Li, H. Zhao, *Chem. Eng. J.* **2024**, *480*, 148014.

[24] M.-D. Zhang, J.-R. Huang, W. Shi, P.-Q. Liao, X.-M. Chen, *J. Am. Chem. Soc.* **2023**, *145*, 2439-2447.
